# Coumarin-Resveratrol-Inspired Hybrids as Monoamine Oxidase B Inhibitors: 3-Phenylcoumarin *versus* *trans*-6-Styrylcoumarin

**DOI:** 10.3390/molecules27030928

**Published:** 2022-01-29

**Authors:** Marco Mellado, César González, Jaime Mella, Luis F. Aguilar, Ismail Celik, Fernanda Borges, Eugenio Uriarte, Giovanna Delogu, Dolores Viña, Maria J. Matos

**Affiliations:** 1 Instituto de Investigación y Postgrado, Facultad de Ciencias de la Salud, Universidad Central de Chile, Santiago 8330507, Chile; marco.mellado@pucv.cl; 2 Departamento de Química, Universidad Técnica Federico Santa María, Valparaíso 2340000, Chile; cesar.gonzalez@usm.cl; 3 Instituto de Química y Bioquímica, Facultad de Ciencias, Universidad de Valparaíso, Valparaíso 2340000, Chile; jaime.mella@uv.cl; 4 Centro de Investigación Farmacopea Chilena (CIFAR), Universidad de Valparaíso, Valparaíso 2340000, Chile; 5 Instituto de Química, Facultad de Ciencias, Pontificia Universidad Católica de Valparaíso, Valparaíso 2340000, Chile; luis.aguilar@pucv.cl; 6 Department of Pharmaceutical Chemistry, Faculty of Pharmacy, Erciyes University, 38000 Kayseri, Turkey; ismailcelik@erciyes.edu.tr; 7 CIQUP, Departamento de Química e Bioquímica, Faculdade de Ciências, Universidade do Porto, 4169-007 Porto, Portugal; fborges@fc.up.pt; 8 Departamento de Química Orgánica, Facultade de Farmacia, Universidade Santiago de Compostela, 15782 Santiago de Compostela, Spain; eugenio.uriarte@usc.es; 9 Instituto de Ciencias Químicas Aplicadas, Universidad Autónoma de Chile, Santiago 7500912, Chile; 10 Department of Life and Environmental Sciences, University of Cagliari, Monserrato, 09042 Cagliari, Italy; delogug@unica.it; 11 Chronic Diseases Pharmacology Group, Center for Research in Molecular Medicine and Chronic Diseases (CIMUS), Universidade de Santiago de Compostela, 15782 Santiago de Compostela, Spain; 12 Departamento de Farmacología, Farmacia y Tecnología Farmacéutica, Universidade de Santiago de Compostela, 15782 Santiago de Compostela, Spain

**Keywords:** 3-phenylcoumarin, *trans*-6-styrylcoumarin, *trans*-resveratrol, monoamine oxidase B inhibitors, 3D-QSAR models, molecular docking, molecular dynamics

## Abstract

Monoamine oxidases (MAOs) are attractive targets in drug design. The inhibition of one of the isoforms (A or B) is responsible for modulating the levels of different neurotransmitters in the central nervous system, as well as the production of reactive oxygen species. Molecules that act selectively on one of the MAO isoforms have been studied deeply, and coumarin has been described as a promising scaffold. In the current manuscript we describe a comparative study between 3-phenylcoumarin (*endo* coumarin-resveratrol-inspired hybrid) and *trans*-6-styrylcoumarin (*exo* coumarin-resveratrol-inspired hybrid). Crystallographic structures of both compounds were obtained and analyzed. 3D-QSAR models, in particular CoMFA and CoMSIA, docking simulations and molecular dynamics simulations have been performed to support and better understand the interaction of these molecules with both MAO isoforms. Both molecules proved to inhibit MAO-B, with *trans*-6-styrylcoumarin being 107 times more active than 3-phenylcoumarin, and 267 times more active than *trans*-resveratrol.

## 1. Introduction

Monoamine oxidases (MAOs) are very interesting and well-characterized targets in drug design and discovery [1]. The inhibition of one of the isoforms (A or B) is responsible for modulating the levels of different neurotransmitters in the central nervous system, as well as the production of reactive oxygen species [2]. Serotonin, norepinephrine, epinephrine and melatonin are mostly degraded by MAO-A, phenethylamine and benzylamine are mostly degraded by MAO-B, and both isoforms mediate the degradation of dopamine, tryptamine and tyramine equally [1,2,3]. Selective MAO inhibitors have been extensively studied [3]. In particular, MAO-B selective compounds may be interesting in the search for new molecules against Parkinson diseases [4], whereas MAO-A inhibitors are selectively used in therapeutics for depression [5]. Computer-aided approaches have been widely used in this field [6]. The design of compounds with selective activity on one of both MAO isoforms has been supported by both quantitative structure–activity relationship (QSAR) and molecular docking techniques [7,8].

Coumarin is a privileged scaffold in both Medicinal Chemistry and Chemical Biology [9,10]. The simplicity and versatility of this scaffold make it very attractive as a building block for more complex structures with a huge variety of applications based on the substitution patterns [11]. Resveratrol is another important scaffold for the design of molecules with a huge range of properties, including a combination of antioxidant and enzymatic inhibition capacities [12]. These compounds have been inspiring medicinal chemists in the design of antioxidants and enzymatic inhibitors for several years [13,14]. 

**Figure 1:**
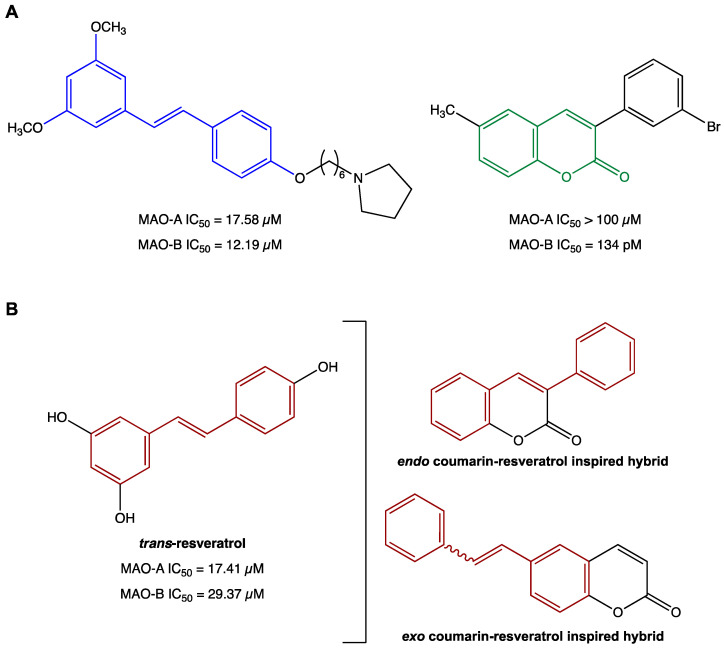
Rationale for the current work. (**A**) Examples of resveratrol and coumarin-inspired compounds with MAO-A and MAO-B activities. The scaffolds are highlighted in blue and green, respectively. (**B**) Chemical structure of *trans*-resveratrol, with MAO-A and MAO-B IC_50_ values, and chemical structures of both studied coumarin-resveratrol-inspired hybrids. *trans*-Stilbene is highlighted in red.

Based on the chemical structures of coumarin and resveratrol, and their pharmacological properties, in our research group we have been working on the development of different coumarin-resveratrol-inspired hybrids. In recent years, our group has been paying special attention to 3-arylcoumarin derivatives (Figure 1), as these molecules contain *trans*-stilbene on their structure [14]. Based on this preliminary data, we decided to design and synthesize two different hybrid molecules: 3-phenylcoumarin (*endo* coumarin-resveratrol-inspired hybrid) and *trans*-6-styrylcoumarin (*exo* coumarin-resveratrol-inspired hybrid). These are the simplest structures containing both coumarin and *trans*-stilbene, being considered the two basic scaffolds, and the main goal of this study is to study and compare them. Both compounds have been studied for their MAO inhibition and compared with *trans*-resveratrol (Figure 1). Crystallographic structures of both compounds have been obtained and analyzed. 3D-QSAR models, in particular CoMFA and CoMSIA, and docking simulations analysis have been performed to support and better understand the interaction of these molecules with both MAO isoforms.

This manuscript is organized in different parts. The details for the synthesis and pharmacological evaluation of the titled molecules are initially presented. Then, the CoMFA and CoMSIA models and docking studies, performed using an internal database of human MAO-A (*h*MAO-A) and human MAO-B (*h*MAO-B) potent inhibitors, are also presented and discussed. These results are the support for the understanding of the last part of the study. The QSAR and docking data together aim to shed light on the interactions of both 3-phenylcoumarin and *trans*-6-styrylcoumarin with the active sites of *h*MAO-A and *h*MAO-B. This information may be useful in the future design and study of coumarin and resveratrol-inspired hybrids as *h*MAO potent and selective inhibitors.

## 2. Results and Discussion

### 2.1. Chemistry

With the aim of finding out the structural features for the MAO inhibitory activity and selectivity, the present manuscript reports the design, synthesis and comparative biological study of 3-phenylcoumarin and *trans*-6-styrylcoumarin, in which two different forms of coumarin-resveratrol-inspired hybridization—*endo* and *exo*—are explored (Figure 1).

**Scheme 1:**
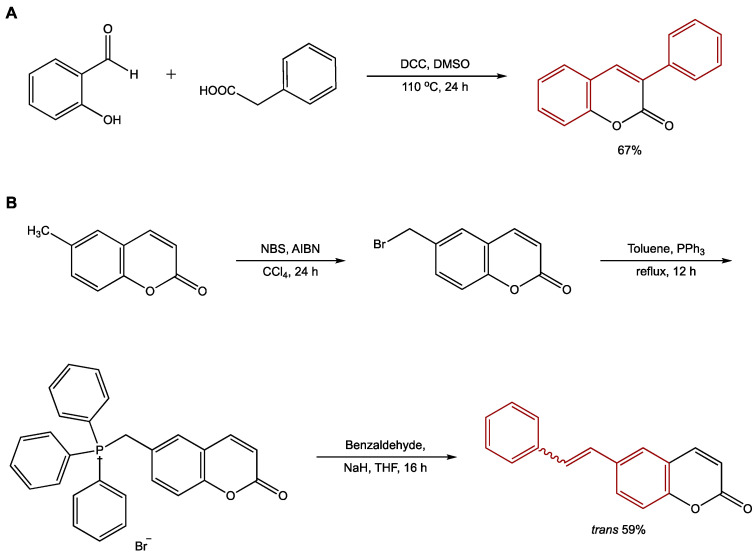
Reactions and general conditions. (**A**) Perkin reaction to obtain 3-phenylcoumarin. (**B**) Bromination of 6-methylcoumarin, synthesis of the phosphonium bromide intermediate, and Wittig reaction to obtain 6-styrylcoumarins (*cis* and *trans* isomers).

3-Phenylcoumarin was prepared according to the protocol described by our research group [15] and the conditions mentioned in Scheme 1A. A Perkin condensation, by reacting the 2-hydroxybenzaldehyde with the phenylacetic acid, in the presence of dicyclohexylcarbodiimide (DCC), in dimethyl sulfoxide (DMSO), gave the desired 3-phenylcoumarin, at a 67% yield. The crystallographic analysis of this molecule has been previously described by our research group [16]. The planarity of the coumarin moiety has been corroborated by analyzing the torsion angles between their carbons. In addition, the angle between the carbons of the coumarin nucleus (C5-C6-C7-C8, Supplementary Materials Figure S1) and the 3-phenyl ring is −47.6° [16]. This value is typical for the torsion permitted by the rotation of this bond [16].

*trans-*6-Styrylcoumarin was prepared according to the protocol described by Cushman et al. [17] and the conditions represented in Scheme 1B. A Wittig reaction of phosphonium bromide with 2-hydroxybenzaldehyde in tetrahydrofuran (THF) and in the presence of sodium hydride (NaH), followed by preparative thin layer chromatographic separation, gave the corresponding *cis/trans*-stilbenes. The diastereomeric *cis*/*trans* ratio of the 6-styrylcoumarin was 30:70. After purification, *trans-*6-styrylcoumarin was obtained at a 59% yield. The phosphonium bromide intermediate was prepared from the 6-bromomethylcoumarin by adding triphenylphosphine (PPh_3_) in toluene. The 6-bromomethylcoumarin was previously prepared by reacting 6-methylcoumarin (commercially available) with *N*-bromosuccinimide (NBS) and azobisisobutyronitrile (AIBN) to obtain the bromomethyl derivative. All the details for both methodologies and compound characterizations are described in the supporting information.

**Figure 2:**
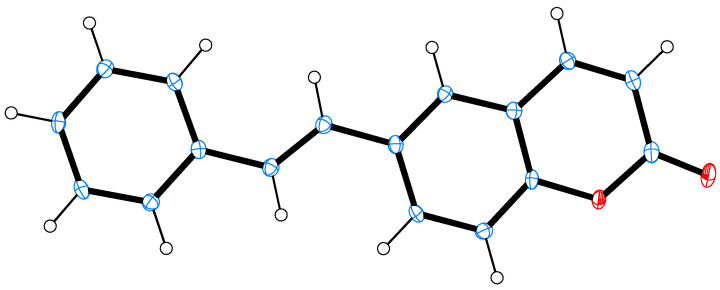
A view of *trans*-6-styrylcoumarin. Displacement ellipsoids are drawn at the 50% level.

*trans*-6-Styrylcoumarin (C_17_H_12_O_2_) is a colorless solid. This stilbene-coumarin hybrid possesses one aromatic ring shared by both parts of the final condensed structure. Because of the double bond of the stilbene part, the final compound can present both *cis* and *trans* configurations. After the purification process, the ^1^H NMR allowed the identification of the isolated *trans* isomer. A double duplet is observed at 7.36 ppm with a coupling constant of 15.5 Hz. The X-ray analysis of this compound (Figure 2) aimed to contribute to the elucidation of the structural requirements needed to understand the geometry of the most abundant stereoisomer, *trans*-6-styrylcoumarin. The *trans* geometry of the derivative, previously described as ^1^H NMR, was confirmed by the crystallographic structure. High-resolution single-crystal X-ray diffraction data was recorded at 100 K. The planarity of both benzenic rings and the pyrone ring is evident from the analysis of the crystallographic data (Figure 2, Supplementary Materials Figures S2 and S3). From the single-crystal diffraction measurements it can be concluded that the experimental bond length is within normal values with the average C_sp_^2^-C_sp_^2^ bond length (Supplementary Materials Figure S2, C11-C12 = 1.338 Å). In addition, the C-7-C11-C12-C13 (Supplementary Materials Figure S2) angle is −176.67°, typical of the *trans* isomer. The planarity of the rings is also evident by the torsion angle values between their carbons. The packing diagram of the structure allows the interpretation of the spatial orientation of the molecules (Supplementary Materials Figure S2). All the crystallographic details are described in the supplementary data (Supplementary Materials Tables S1–S6).

### 2.2. Pharmacology

In vitro effects of the synthesized molecules on the activity of both *h*MAO isoforms have been evaluated using an Amplex^®^ Red MAO assay kit and recombinant *h*MAO isoenzymes expressed in baculovirus-infected BTI insect cells (Sigma Aldrich) [15]. The results for both *h*MAO-A and *h*MAO-B inhibitory activities are presented in Table 1.

In order to compare the activity of the new derivatives with reference compounds, *trans*-resveratrol, selegiline and iproniazid were tested. *Trans*-resveratrol is structurally similar to the synthesized compounds. Selegiline is an irreversible MAO-B inhibitor used alone to treat early Parkinson’s disease or with levodopa for treatment of advanced Parkinson’s disease. Iproniazid is a hydrazine, non-selective and irreversible MAO inhibitor, used as an antidepressant for several years in several countries. Therefore, these molecules have been selected as reference compounds for this study. The three reference compounds display activity against both MAO-A and MAO-B isoforms, with selegiline being much more active against MAO-B than MAO-A (SI = 3362.5). *trans*-Resveratrol and iproniazid are considered non-selective inhibitors (SI = 0.59 and 0.87, respectively). 

Interestingly, both synthetic compounds (3-phenylcoumarin and *trans*-6-styrylcoumarin) proved to be *h*MAO-B selective inhibitors, with *trans*-6-styrylcoumarin being 107 times more active than 3-phenylcoumarin, and 267 times more active than *trans*-resveratrol. Compared to resveratrol derivatives described by Kong et al. [13], 3-phenylcoumarin proved to display similar activity on MAO-B, being selective for this isoform. *trans*-6-Styrylcoumarin proved to be 110 times more active than the best compound of that series, also being a MAO-B selective inhibitor. Finally, Santana et al. [14] described different 3-arylcoumarin derivatives that have been optimized during the past decade, finding molecules displaying outstanding MAO-B activity and selectivity, such as the 3-(3′-bromophenyl)-6-methylcoumarin, with a MAO-B IC_50_ of 134 pM. The characteristics and substitution pattern of this molecule may be the inspiration for further modifying the *trans*-6-styrylcoumarin scaffold.

This information may be useful in the design and synthesis of new series of coumarin-resveratrol-inspired hybrids. To better understand this experimental data, computational studies were carried out, using an internal database of molecules. 3D-QSAR models and docking simulations were performed, and the results from these simulations analyzed.

### 2.3. Computer-Aided Studies Using an Internal Database of Coumarins and Chromone Analogs

#### 2.3.1. D-QSAR Studies

The 3D-QSAR studies (CoMFA and CoMSIA models) were developed according to the procedure previously reported by our research group [8], using Sybyl X software (Tripos Inc., St. Louis, MO, USA). The data set used for the CoMFA and CoMSIA models were formed by 30 compounds presenting *h*MAO-A inhibitory activity and 157 compounds presenting *h*MAO-B inhibitory activity (6 selective MAO-A inhibitors, 133 selective MAO-B inhibitors and 24 non-selective MAO-A/MAO-B inhibitors), as previously published by our research group (Supplementary Materials Figure S4, Tables S7 and S8) [8,18]. To develop the models that relate the chemical structure and the inhibitory activity of differently substituted coumarins on MAO-A and MAO-B, an in-depth QSAR study of a wide variety of coumarins was carried out. The structures selected for this study present chemical similarity to the coumarin-resveratrol-inspired hybrids under study. Among the studied structures with inhibitory activity against both isoforms, 3-arylcoumarins, 3-amidocoumarins, 3-aminocoumarins, 3-arylbenzo[*f*]coumarins and chromone analogs are included (Supplementary Materials Table S7) [15,19,20,21,22,23,24,25,26,27,28,29,30,31,32,33,34]. In the case of MAO-B, the compounds showed a pIC_50_ range of five logarithmic units, while in the case of MAO-A, the range was two logarithmic units. From the selected database, some compounds must be highlighted for their selectivity and activity against MAO-B isoform. Compounds **5** [3-(3′-methoxyphenyl)-6-methylcoumarin], **17** [3-(4′-methylphenyl)-6-methylcoumarin] and **64**–**67** [3-(4′-bromophenyl)-6-methylcoumarin, 3-(3′-bromophenyl)-6-methylcoumarin, 3-(4′-bromophenyl)-6-mehtoxycoumarin and 3-(3′-bromophenyl)-6-methoxycoumarin] display activities in the picomolar range. All these compounds present methyl, methoxy or bromine substituents on the 3-phenylcoumarin scaffold. In particular, positions 6 and 3′ or 4′ are the best spots to potentiate MAO-B activity and selectivity. To obtain the best CoMFA and CoMSIA models, a systematic search was carried out through the combination of different properties (steric, electrostatic, hydrophobic, and hydrogen bond donors and acceptors, Tables S9 and S10), looking for the models that present high statistical values, particularly those models with a value of *q*^2^ > 0.5 [35,36].

The best CoMFA models for MAO-A and MAO-B presented *q*^2^ values of 0.612 and 0.841, respectively. While the best CoMSIA models for MAO-A and MAO-B presented *q*^2^ values of 0.523 and 0.772, respectively (Supplementary Materials Figure S6). Therefore, the CoMFA models proved to have a better internal predictive capacity than the CoMSIA models. On the other hand, the external predictive capacity of the models was assessed through the calculation of the *r*^2^*_test_*. The models presented similar characteristics, obtaining r^2^ values for the test set for MAO-A and MAO-B CoMFA of 0.805 and 0.808, respectively. In the case of MAO-A and MAO-B CoMSIA models, the r^2^ values were 0.853 and 0.911, respectively. Therefore, the external predictive capacity is slightly higher for CoMSIA models.

For the visualization of the obtained contour maps, the compounds that exhibited the highest activity on each MAO isoform have been selected. In the case of MAO-A, 3-(3′,4′-dihydroxyphenyl)benzo[*f*]coumarin (**84**, pIC_50_ = 6.2441) was analyzed, while to project the results for the MAO-B isoform, 3-(3′-bromophenyl)-6-methylcoumarin (**65**, pIC_50_ = 9.8729) was selected. The results for the MAO-A enzyme are not shown here; all the results are included in the supporting information (Supplementary Materials Figure S7).

##### Steric Map of MAO-B Inhibitors

**Figure 3:**
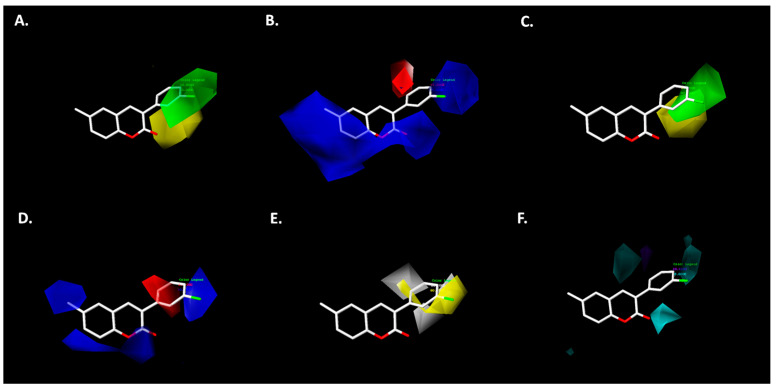
Contour map of 3D-QSAR results for MAO-B inhibitors around 3-(3′-bromophenyl)-6-methylcoumarin (**65**). (**A**) CoMFA steric map. (**B**) CoMFA electrostatic map. (**C)** CoMSIA steric map. (**D**) CoMSIA electrostatic map. (**E**) CoMSIA hydrophobic map. (**F**) Hydrogen-bond donor map. Color code: steric contour map green—bulky groups are favorable for the activity; steric contour map yellow—small groups are favorable for the activity; electrostatic contour map red—negative charge is favorable for the activity; electrostatic contour map blue—positive charge is favorable for the activity; hydrophobic contour map yellow—hydrophobic groups are favorable for the activity; hydrophobic contour map grey—hydrophilic groups are favorable for the activity; hydrogen-bond donor map cyan—hydrogen-bond donor groups are favorable for the activity; hydrogen-bond donor map purple—hydrogen-bond donor groups are unfavorable.

The CoMFA and CoMSIA steric maps analysis shows a great similarity between them (Figure 3A–C). This map shows a green polyhedron projecting into *ortho*, *meta* and *para* substitutions of the aryl ring attached to position 3 of the coumarin scaffold, indicating that bulky substituents at this position may favor the MAO-B inhibitory activity. This is consistent with our previous data, especially for both *meta* and *para* positions, and the activities shown by 3-arylcoumarins bearing substituents as methyl, hydroxyl, methoxy, nitro, amine or bromine (e.g., compounds **2**–**5**, **9**, **10**, **12**–**14**, **17**–**21**, **25**, **32**, **33**, **37**–**39**, **52**–**54**, **64**–**69**, **73**–**76**, **78**, **87**, **88**, **90**, **91**, **137** and **141**). In addition, on the back of the green polyhedron, there is a yellow polyhedron, which indicates that bulky substituents cause a decrease in the MAO-B inhibitory activity. This phenomenon is directly related to the compounds that present a “spacer” between the coumarin scaffold and the aryl ring (e.g., amide or carbamate), providing rotatable bonds which orient the aryl ring towards this area, presenting the compounds MAO-B pIC_50_ < 6.5 (e.g., compounds **42**–**48**, **60**, **92**–**96**, **109**, **115**, **128** and **135**).

##### Electrostatic Map of MAO-B Inhibitors

The electrostatic maps analysis shows similarities for the coumarin scaffold and the aryl fragment linked to position 3 (Figure 3B–D). In the first case, a blue polyhedron is projected into the carbonyl carbon, indicating that the electron-deficient characteristic of this group is necessary for the MAO-B inhibitory activity. In addition, a blue polyhedron is projected near positions 6, 7 and 8, indicating that electron-deficient substituents at these positions cause an increase in the MAO-B inhibitory activity. This characteristic is consistent with the presence of 6-methyl and 8-methyl substituents, which project the deficient part of electrons on this polyhedron, causing the high activity of these compounds (e.g., compounds **1**–**6**, **8**–**10**, **12**–**14**, **17**–**21**, **24**, **25**, **36**–**39**, **64**, **65**, **87**, **88**, **90**, **91**, **137** and **140**). On the other hand, in the case of the aryl group linked to position 3 of the coumarin scaffold, a red polyhedron is projected in the *ortho* position, showing that a group rich in electrons may favor the MAO-B inhibitory activity. Thus, some compounds presenting methoxy or hydroxyl groups, or bromine atoms at this position, showed pIC_50_ > 6.5 (e.g., compounds **6**, **8** and **140**). Additionally, a blue polyhedron is projected in the *meta* position, indicating that an electron-deficient substituent may favor the activity. This is consistent with the presence of methyl ether and nitro substituents (e.g., compounds **25** and **89**).

##### Hydrophobic Map of MAO-B Inhibitors

The hydrophobic map analysis shows that the main modifications affecting the activity are found in the aryl ring linked to position 3 of the coumarin scaffold (Figure 3E). In this map, in the *meta* position of the aryl ring, a yellow polyhedron is projected, indicating that a hydrophobic substituent may cause an increase in the MAO-B inhibitory activity, concordant with the activity of compounds **3**–**5**, **10**, **12**, **18**–**21**, **65**, **67**, **69**, **74, 76**, **78**, **137**, **140** and **141**, that have bromine, methyl or methoxy substituents (pIC_50_ > 6.5). Additionally, in the *ortho* and *para* positions of the aryl ring, white polyhedra are projected, indicating that hydrophilic substituents (e.g., hydroxyl or amine) may cause an increase in the activity (pIC_50_ > 6.5). This is observed for compounds **24** and **91**.

##### Hydrogen-Bond Donor Map of MAO-B Inhibitors

The hydrogen-bond donor map analysis shows that close to position 4 of the coumarin scaffold, a cyan polyhedron is projected, indicating that substituents with hydrogen-bond donor characteristics may favor the MAO-B inhibitory activity (Figure 3F). This is observed for hydroxyl substituents, such as in the case of compound **24**, with a pIC_50_ < 6.5. Furthermore, when a “spacer” is introduced between the coumarin scaffold and an aryl ring, it is observed that the amide linker can orient the nitrogen atom towards a cyan polyhedron, favoring the MAO-B inhibitory activity of these compounds. This is observed for compounds **98**–**108**, **110**–**114**, **116**–**127** and **129**–**134**. In the *para* position of the same aryl ring, a cyan polyhedron is also projected. This is consistent with the high MAO-B inhibitory activity of compound **91**, presenting a nitrogen atom at this position. However, this property can also be extended to halogen atoms (e.g., chlorine and bromine), which can experience properties like hydrogen-bonds, as the case of compounds **20**, **39**, **64**, **66**, **73**, **106**, **114**, **121**, **127** and **134** (pIC_50_ > 6.5). Additionally, in the *ortho* position of this ring, a purple polyhedron is projected, indicating that a hydrogen-bond donor group may decrease the MAO-B inhibitory activity, as is the case for compound **89** (pIC_50_ = 5.664).

#### 2.3.2. Molecular Docking

**Figure 4:**
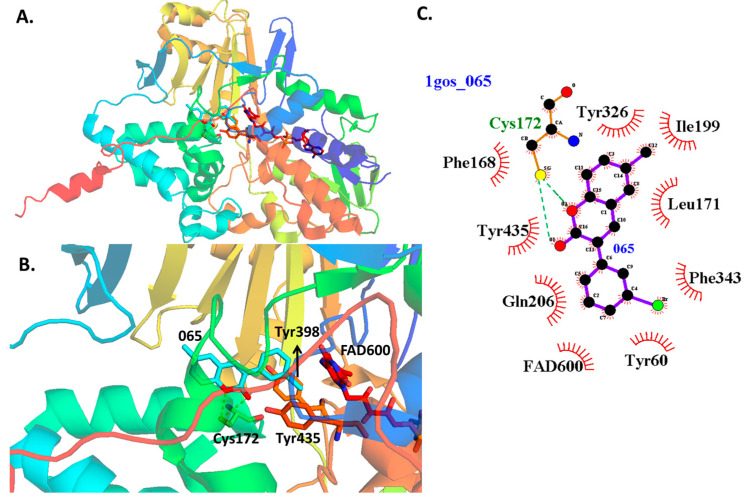
Molecular docking of 3-(3′-bromophenyl)-6-methylcoumarin (**65**) on the MAO-B binding pocket. (**A**) 3D overview within the MAO-B active site. (**B**) Best orientation of compound **65** on the MAO-B active site. (**C**) Details of polar, *van der Waals* and hydrogen bonding interactions established by compound **65** with the residues on the MAO-B active site.

To complement the CoMFA and CoMSIA studies, molecular docking using the best compounds from the database [3-(3′,4′-dihydroxyphenyl)benzo[*f*]coumarin **84** for MAO-A and 3-(3′-bromophenyl)-6-methylcoumarin **65** for MAO-B] was performed. MAO-A (PDB ID = 2Z5Y) [37] and MAO-B (PDB ID = 1GOS) [38] crystal structures, and Autodock vina [39] software, were used in this study. PyMOL [40] and LigPlot + [41,42] were used to visualize the results, according to what had been reported by our research group [8,18,43]. The results for the MAO-A enzyme are not shown here; all the results are included in the supporting information (Supplementary Materials Figure S8). For MAO-B (Figure 4), it is observed that the 3-bromophenyl ring of compound **65** is positioned between the Tyr435 and Tyr398 residues, in addition to being perpendicular to the FAD600 residue, favoring the polarization of the ligand to be oxidized according to the reaction mechanism described by Abad et al. [44]. Complementing this information with the CoMFA and CoMSIA contour maps analysis (Figure 3A–C), bulky substituents close to the *ortho* position of the 3-bromophenyl fragment are unfavorable for the MAO-B inhibitory activity, due to the potential blocking of the interaction between the ligand and the Tyr435 and Tyr398 residues, in addition to the hydrophobic and polar repulsions with the Gln206 and Phe343 residues. In addition, the electrostatic map (Figure 3B–D) shows that a group rich in electrons in the *ortho* position of the 3-bromophenyl ring may favor the MAO-B inhibitory activity because it can improve the polar interactions with both Phe343 and Gln206 residues. In this same fragment, close to the 3-bromine substituent, it is indicated that an electron-deficient substituent may favor the MAO-B inhibitory activity, since the polarization of the ligand between the Tyr435 and Tyr398 residues would be more effective. In the case of the coumarin scaffold, it is shown that an electron-poor substituent linked to positions 6, 7, and/or 8 may favor the MAO-B inhibitory activity, which is related to a better polar interaction between this fragment and the Tyr326 and Cys172 residues. The hydrophobic map (Figure 3E) shows that a hydrophobic group close to the 3-bromine of the phenyl fragment may favor the MAO-B inhibitory activity, which is related to the hydrophobic interactions with the aryl fragments of the Tyr435 and Tyr398 residues. Whereas, in the *ortho* position of the same ring, it is expected that the MAO-B inhibitory activity may be favored by the presence of a hydrophilic group, which is directly related to the proximity of both Gln206 and Phe343 residues, favoring the polar interactions with Phe343 and a hydrogen-bond with Gln206. Finally, the hydrogen-bond donor map (Figure 3F) shows that a group of these characteristics in the *ortho* position of the 3-bromophenyl fragment may favor the MAO-B inhibitory activity, related to the potential stabilization of the ligand in the active site by forming a hydrogen-bond with Gln206. Furthermore, close to the carbonyl group of the coumarin scaffold, a hydrogen-bond acceptor is expected to promote the inhibitory activity. This is because Cys172 and Tyr326 residues can participate as hydrogen-bond donors, causing ligand stability within the MAO-B active site.

### 2.4. Computational Studies for the New Coumarin-Resveratrol-Inspired Hybrids

Based on the previous computer-aided study (CoMFA, CoMSIA and molecular docking) on a series that includes 3-arylcoumarins, and due to the structural similarity of these compounds with the polyphenol resveratrol, two coumarin-resveratrol-inspired hybrids (Figure 1) were designed, synthesized, their inhibitory activity on both *h*MAO-A and *h*MAO-B studied (Table 1), and their interactions with both isoenzymes studied by computational approaches. The results of the experimental pIC_50_, and the calculated values using the developed models, are found in Table 2 and Table 3.

**Figure 5:**
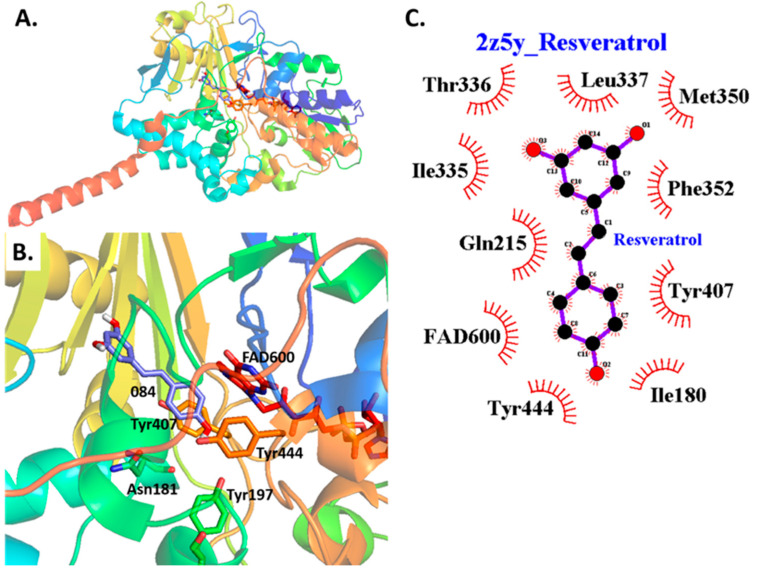
Molecular docking of *trans*-resveratrol on the MAO-A binding pocket. (**A**) 3D overview within the MAO-A active site. (**B**) Best orientation of *trans*-resveratrol on the MAO-A active site. (**C**) Details of polar and *van der Waals* interactions established with the residues on the MAO-A active site.

Considering the only active compound in Table 2 on MAO-A and projecting the *trans*-resveratrol structure on the field maps obtained from the CoMFA and CoMSIA analysis (Supplementary Materials Figures S9 and S10, respectively), the *trans*-resveratrol projects the 4-hydroxyphenyl group (a bulky substituent) towards the green polyhedron, favoring the MAO-A inhibitory activity. Additionally, when superimposing the *trans*-resveratrol on the electrostatic map, it can be appreciated that the 4-hydroxyphenyl group is oriented towards the red polyhedron, indicating that the electron-rich characteristic of this ring may favor the MAO-A inhibitory activity. The projection of the 3-hydroxyl group (of the 3,5-dihydroxyphenyl fragment of the *trans*-resveratrol) in another red polyhedron may cause the same effect. When comparing the alignment of the *trans*-resveratrol obtained in the 3D-QSAR study, and the interaction of compound **84** and *trans*-resveratrol with the MAO-A active site analyzed by docking (Figure 5), it can be observed that the 3,5-dihydroxyphenyl group is oriented towards the Met350 and Thr336 residues, favoring polar and *van der Waals* interactions, while the residues Leu337 and Ile335 allow for stabilization by hydrophobic interactions. In addition, the 4-hydroxyphenyl group of the *trans*-resveratrol is oriented towards the *FAD600*, and between the Tyr444 and Tyr407 residues, allowing the polarization of the ligand for oxidation [44].

Table 3 shows the inhibition values (pIC_50_) obtained experimentally, and the calculated values of 3-phenylcoumarin, *trans*-6-styrylcoumarin and *trans*-resveratrol, with a good correlation between the experimental and calculated values, according to the CoMFA and CoMSIA models (Res < 1.0).

**Figure 6:**
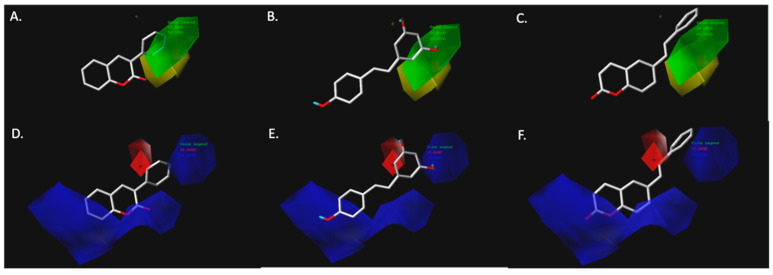
Overlay of *trans*-resveratrol, 3-phenylcoumarin and *trans*-6-styrylcoumarin on the contour map obtained from the MAO-B CoMFA model. (**A**) *Trans*-resveratrol and steric map. (**B**) 3-Phenylcoumarin and steric map. (**C**) *trans*-6-Styrylcoumarin and steric map. (**D**) *Trans*-resveratrol and electrostatic map. (**E**) 3-Phenylcoumarin and electrostatic map. (**F**) *trans*-6-Styrylcoumarin and electrostatic map. Color code: steric contour map green—bulky groups are favorable for the activity; steric contour map yellow—small groups are favorable for the activity; electrostatic contour map red—negative charge is favorable for the activity; electrostatic contour map blue—positive charge is favorable for the activity; hydrophobic contour map yellow—hydrophobic groups are favorable for the activity; hydrophobic contour map grey—hydrophilic groups are favorable for the activity.

**Figure 7:**
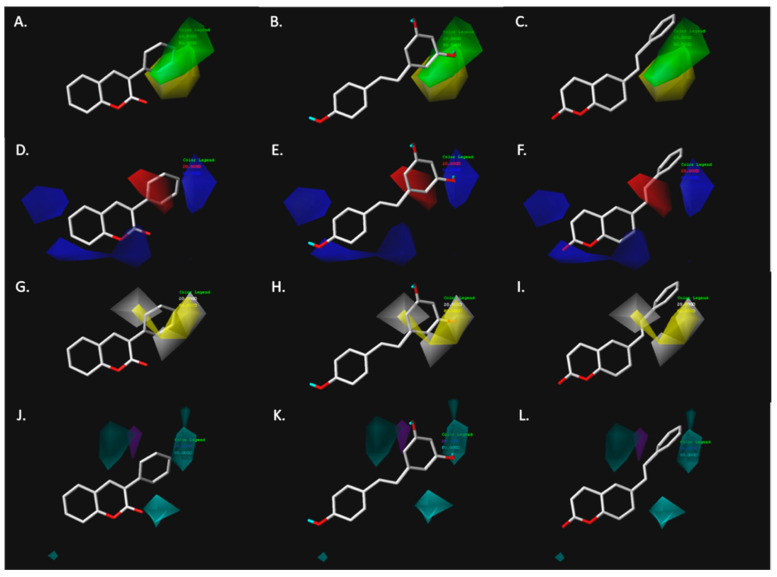
Overlay of 3-phenylcoumarin, *trans*-resveratrol and *trans*-6-styrylcoumarin on the contour map obtained from the MAO-B CoMSIA model. (**A**) *trans*-Resveratrol and steric map. (**B**) 3-Phenylcoumarin and steric map. (**C**) *trans*-6-Styrylcoumarin and steric map. (**D**) *trans*-Resveratrol and electrostatic map. (**E**) 3-Phenylcoumarin and electrostatic map. (**F**) *Trans*-6-styrylcoumarin and electrostatic map. (**G**) *trans*-Resveratrol and hydrophobic map. (**H**) 3-Phenylcoumarin and hydrophobic map. (**I**) *Trans*-6-styrylcoumarin and hydrophobic map. (**J**) *trans*-Resveratrol and hydrogen-bond donor map. (**K**) 3-Phenylcoumarin and hydrogen-bond donor map. (**L**) *trans*-6-Styrylcoumarin and hydrogen-bond donor map. Color code: steric contour map green—bulky groups are favorable for the activity; steric contour map yellow—small groups are favorable for the activity; electrostatic contour map red—negative charge is favorable for the activity; electrostatic contour map blue—positive charge is favorable for the activity; hydrophobic contour map yellow—hydrophobic groups are favorable for the activity; hydrophobic contour map grey—hydrophilic groups are favorable for the activity; hydrogen-bond donor map cyan—hydrogen-bond donor groups are favorable for the activity; hydrogen-bond donor map purple—hydrogen-bond donor groups are unfavorable.

**Figure 8:**
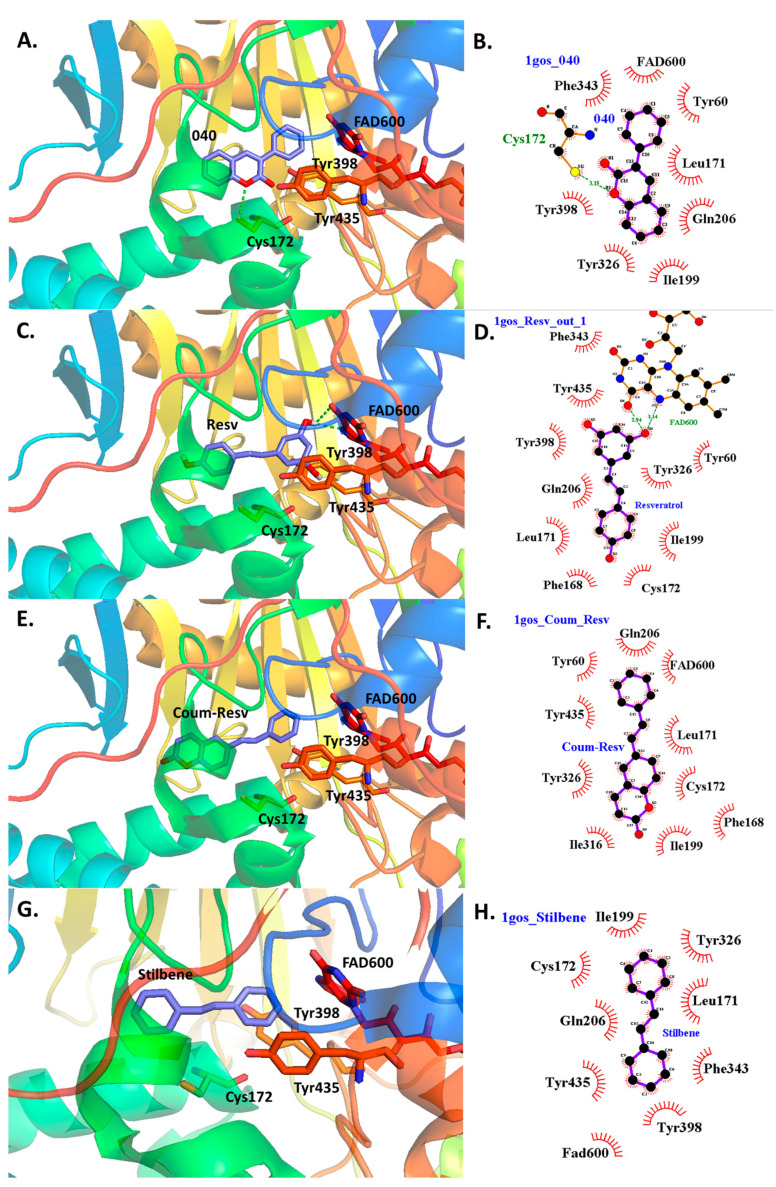
Molecular docking of 3-phenylcoumarin, *trans*-resveratrol, *trans*-6-styrylcoumarin and *trans*-stilbene on MAO-B binding pocket. (**A**) Best orientation of 3-phenylcoumarin on the MAO-B active site. (**B**) Details of polar and *van der Waals* interactions established by 3-phenylcoumarin with the residues of the MAO-B active site. (**C**) Best orientation of *trans*-resveratrol on the MAO-B active site. (**D**) Details of polar and *van der Waals* interactions established by *trans*-resveratrol with the residues of the MAO-B active site. (**E**) Best orientation of *trans*-6-styrylcoumarin on the MAO-B active site. (**F**) Details of polar and *van der Waals* interactions established by *trans*-6-styrylcoumarin with the residues of the MAO-B active site. (**G**) Best orientation of *trans*-stilbene on the MAO-B active site. (**H**) Details of polar and *van der Waals* interactions established by *trans*-stilbene with the residues of the MAO-B active site.

When making the superposition of the evaluated structures on the contour maps obtained from the CoMFA and CoMSIA models (Figure 6 and Figure 7, respectively), there is an agreement between the values of the experimental activity and the ones predicted by the models (Res < 1.00). In the case of the 3-phenylcoumarin, it can be observed that the phenyl ring is oriented towards the green polyhedron (steric map), indicating that a bulky group may favor the MAO-B inhibitory activity, while the carbonyl group of the coumarin scaffold is projected near a blue polyhedron (electrostatic map), indicating that an electron-rich substituent may decrease the MAO-B inhibitory activity. Complementing this information with the data obtained from the molecular docking (Figure 8), it is evidenced that the phenyl ring of the 3-phenylcoumarin is positioned between the Tyr398 and Tyr435 residues, and perpendicular to the FAD600. It is also appreciated that there is the formation of a hydrogen-bond between the oxygen atom of the cyclic ester and the Cys172 residue, an interaction that stabilizes the ligand within the MAO-B active site. All these interactions, in addition to the hydrophobic ones (Figure 8B), produce the calculated affinity energy of −7.6 kcal/mol.

In the case of the *trans*-resveratrol, the 3,5-dihydroxyphenyl ring is oriented towards the green polyhedron (Figure 6 and Figure 7, steric map), indicating that a bulky group favors the MAO-B inhibitory activity. In the electrostatic map, the 3-hydroxyl group is oriented towards a blue polyhedron, indicating that the presence of an electron-poor group favors the MAO-B inhibitory activity due to the deficiency of electrons. Complementing this information with that obtained from the molecular docking analysis, it is evident that the 3,5-dihydroxyphenyl ring is oriented between the Tyr398 and Tyr435 residues, and perpendicular to the FAD600. In addition, the 3-hydroxy group of the 3,5-dihydroxyphenyl ring generates two hydrogen-bonds with the FAD600, stabilizing the ligand within the MAO-B active site. This is consistent with both the presence of the previously mentioned blue polyhedron and the cyan polyhedron of the hydrogen-bond donor map (Figure 7). These interactions, in addition to the hydrophobic ones (Figure 8D), produce the calculated affinity energy of −7.1 kcal/mol.

The most favorable alignment for the *trans*-6-styrylcoumarin on the MAO-B isoform corresponds to the alignment of the stilbene as the pharmacophoric core, with an affinity energy of −8.3 kcal/mol. The 6-styryl fragment linked to the coumarin scaffold is oriented towards the Tyr398 and Tyr435 residues, and perpendicular to the FAD600, with the stability of the ligand being generated mainly by the polar and hydrophobic interactions. Contrasting this information with that of the contour maps obtained from the CoMFA and CoMSIA models (Figure 6 and Figure 7), it can be observed that around the 6-styryl fragment, a voluminous substituent (steric map) may favor the activity, which may be related to the hydrophobic and/or polar interactions with the Tyr60, Gln206 and Tyr435 residues. In the case of the electrostatic map, the 6-styryl fragment is projected into a blue polyhedron, indicating that a substituent poor in electrons may favor the MAO-B inhibitory activity, consistent with the proximity to the Tyr435 and Tyr398 residues, forming polar interactions. Furthermore, in the case of the coumarin scaffold, a blue polyhedron is projected near the carbonyl group, which may be related to the proximity of both Cys172 and Phe208 residues, and their eventual polar interactions, causing the stabilization of the ligand within the MAO-B active site. The hydrophobic map shows that close to the double bond of the 6-styryl fragment, a yellow polyhedron is projected forward (hydrophobic substituents favor the activity) and a white polyhedron backward (hydrophilic substituents favor the activity), which is related to the proximity to the Leu171 and Tyr435 residues, respectively. Finally, the hydrogen-bond donor map shows that close to the phenyl ring of the 6-styryl fragment, a cyan polyhedron is projected (hydrogen bond acceptor substituent favors activity), which is directly related to the proximity of the Tyr435 and Tyr398 residues, key to the polarization of the ligand to be oxidized by the FAD600, as mentioned before. At positions 5 and 7 of the coumarin scaffold, a cyan polyhedron is projected, which is related to the proximity of the Tyr326 and Cys172 residues, respectively. This characteristic would allow these residues to generate hydrogen-bonds with the ligand, stabilizing it within the MAO-B active site.

Complementarily, the interaction between the stilbene scaffold and the MAO-B active site was analyzed (Figure 8G–H), and it was found that one of its aromatic rings is oriented between the Tyr398 and Tyr435 fragments, similar to *trans*-resveratrol. However, the calculated affinity energy is −7.8 kcal/mol, which is lower than the affinity energy calculated for the *trans*-resveratrol, evidencing that one of the hydroxy groups of the 3,5-dihydroxy fragment is important for the stabilization of the ligand within the active site by forming a hydrogen-bond with the FAD600. Moreover, when *trans*-stilbene and *trans-*6-styrylcoumarin are compared, the same orientation can be observed. However, the affinity energies are different between these two compounds (−7.8 kcal/mol and −8.3 kcal/mol, respectively). These differences are related to the hydrophobic and *van der Waals* interactions between the pyrone-core with residues Tyr326, Tyr316 and Phe168*,* which may improve the *trans*-6-styrylcoumarin stabilization into the MAO-B active site.

**Figure 9:**
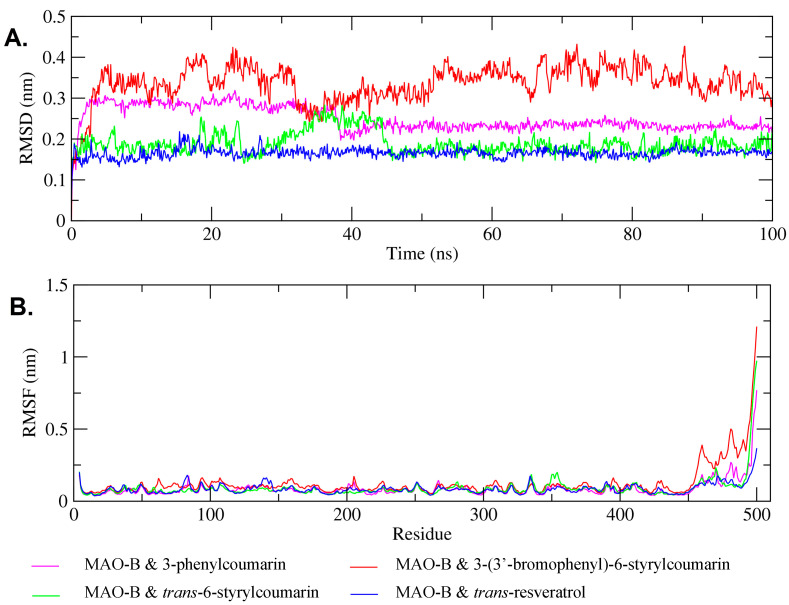
Trajectory analysis of the MD simulation of complexes of MAO-B with 3-phenylcoumarin, 3-(3′-bromophenyl)-6-methylcoumarin, *trans*-6-styrylcoumarin and *trans*-resveratrol. (**A**) Plots of root mean square deviation (RMSD), and (**B**) root mean square fluctuation (RMSF) over 100 ns of protein–ligand complexes.

Molecular dynamics (MD) simulations can be used to study the dynamical movements of macromolecules such as protein, DNA and RNA, drugs, newly designed compounds, natural compounds and endogenous substances naturally found in the human body [45,46]. In this study, 100 ns MD simulations were performed to analyze the stability of 3-phenylcoumarin, 3-(3′-bromophenyl)-6-methylcoumarin, *trans*-6-styrylcoumarin and *trans*-resveratrol protein–ligand complexes obtained from molecular docking studies and to reveal their thermodynamic behavior. Topologies of protein and ligands were performed using Charmm36 force fields. The RMSD, which is calculated according to the protein backbone atoms, is the parameter that expresses the shifts in the protein structure. A stable and low RMSD value indicates that the protein structure is balanced. As shown in Figure 9A, after the first 10 ns of pre-MD simulation, the MAO-B– 3-phenylcoumarin complex proved to remain stable below 0.3 nm, the MAO-B–3-(3′-bromophenyl)-6-methylcoumarin complex proved to remain stable below 0.4 nm, and both MAO-B–*trans*-6-styrylcoumarin and MAO-B–*trans*-resveratrol complexes proved to remain stable around 0.2 nm. RMSF measurements are used to explain the flexibility and movements in protein structure. It is expected that the protein will be more stable and a smaller RMSF value will be measured, with the interactions of the ligand at the active site of the protein. As shown in Figure 9B, for 3-phenylcoumarin, *trans*-6-styrylcoumarin and *trans*-resveratrol protein–ligand complexes that form H bonds with *Tyr435* at the MAO-B active site, the RMSF values are lower than for the 3-(3′-bromophenyl)-6-methylcoumarin complex.

**Figure 10:**
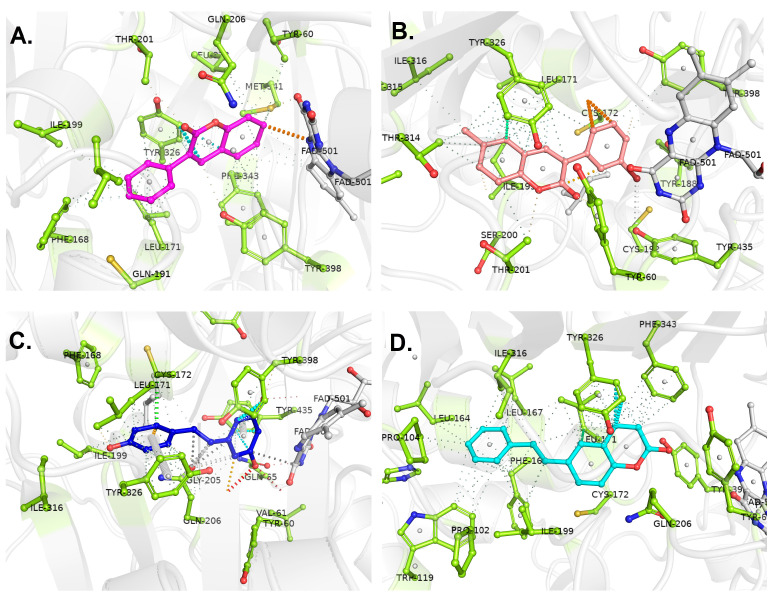
Binding poses of 3-phenylcoumarin (**A**), 3-(3’-bromophenyl)-6-methylcoumarin (**B**), *trans*-resveratrol (**C**) and *trans*-6-styrylcoumarin (**D**) at the active site of MAO-B of 100 ns MD simulations.

Binding poses, at 100 ns, were analyzed to examine the changes that occur with MD simulations of protein–ligand complexes, and their stability at the MAO-B active site. The 2D schematic of the four protein–ligand complex interactions is shown in Supplementary Materials Figure S11. Figure 10A shows the interactions of 3-phenylcoumarin at 100 ns, presenting similar interactions to the molecular docking pose. The interactions with Tyr326 (Pi-Pi T-shaped), Tyr398 and FAD (*van der Waals*), are still present. As shown in Figure 10B, 3-(3′-bromophenyl)-6-methylcoumarin maintained the hydrophobic interactions with Tyr326 (Pi-Pi T-shaped), Ile199, Cys172 and FAD (Pi-alkyl). As shown in Figure 10C, while *trans*-resveratrol formed one H bond with *Tyr435* at the beginning of the MD simulations, two H bonds with FAD and Gly205, and Pi-Pi T-shaped interactions with Tyr326 and Tyr398, were formed at the end of 100 ns. As observed in Figure 10D, the interactions of the coumarin ring of the *trans*-6-styrylcoumarin with Leu171 and Cys172 (Pi-Pi T-shaped and Pi-alkyl) were preserved at the beginning and end of the MD simulations.

Another way to analyze the stability of protein–ligand interactions in MD simulations is to measure the binding free energy between the protein and ligand over time. For this purpose, the binding free energy Molecular Mechanics Poisson–Boltzmann Surface Area (MMPBSA) between 80 and 100 ns was obtained from the sum of the *van der Waals*, electrostatic, polar solvation and SASA energies. As shown in Table 4, *trans*-resveratrol formed the highest binding free energy with a value of −98.903 kJ/mol. 3-Phenylcoumarin and *trans*-6-styrylcoumarin proved to display similar MMPBSA values, −62.139 kJ/mol and −61.865 kJ/mol, respectively. 3-(3′-Bromophenyl)-6-methylcoumarin exhibited the lowest interaction with a value of −26.179 kJ/mol.

In conclusion, the four compounds remained stable at the MAO-B active site according to RMSD, RMSF and MMPBSA analysis from the MD simulations. However, according to the trajectory analysis and binding free energy measurements, the highest interaction is observed for the *trans*-resveratrol, and the lowest interaction is observed for the 3-(3′-bromophenyl)-6-methylcoumarin. According to RMSD and MMPBSA, 3-phenylcoumarin and *trans*-6-styrylcoumarin produced similar potent interactions with MAO-B.

## 3. Conclusions

Four different QSAR models were carried out, presenting high *q*^2^ (0.612, 0.841, 0.523 and 0.772) and *r*^2^*_test_* (0.805, 0.808, 0.853 and 0.911) values. The steric and hydrophobic properties contributed to explaining the activity of the compounds included in the study on both MAO-A and MAO-B enzymes. With the aim of studying and comparing the MAO-B selective activity, two different coumarin-resveratrol-inspired hybrids were synthesized and evaluated. *Trans*-6-styrylcoumarin (the *exo* hybrid compound) proved to be 107 times more potent on this enzyme than 3-phenylcoumarin (the *endo* hybrid compound), and 267 times more active than *trans*-resveratrol, used as a control. *trans*-6-Styrylcoumarin showed a very high selectivity for the MAO-B isoform (pIC_50_ = 6.959). Docking simulations corroborated this information by shedding light on the potential interactions of both molecules with both MAO-A and MAO-B binding pockets. The high MAO-B activity and selectivity of both 3-phenylcoumarin and *trans*-6-styrylcoumarin may be explained by the interaction with the Cys172 residue, replaced in MAO-A binding pocket by the ASN181. Analyzing the MD simulations, all the studied compounds proved to remain stable at the MAO-B active site (RMSD, RMSF and MMPBSA analysis). According to RMSD and MMPBSA, 3-phenylcoumarin and *trans*-6-styrylcoumarin produced similar potent interactions with MAO-B. As the main conclusion, the combination of CoMFA and CoMSIA models (with predictions yielding residuals less than 0.3) and molecular docking can be useful for the future design and synthesis of new families of potent and selective hybrid compounds.

## Figures and Tables

**Table 1:** Experimental *h*MAO-A and *h*MAO-B IC_50_ values of the studied compounds and *trans*-resveratrol (reference inhibitor), using recombinant *h*MAO isoforms expressed in baculovirus-infected BTI insect cells (BTI-TN-5B1-4), and *h*MAO-B selectivity index.

Compounds	IC_50_ hMAO-A (µM)	IC_50_ hMAO-B (µM)	S.I. *
3-Phenylcoumarin	>100	11.81	>8.47
*trans*-6-Styrylcoumarin	>100	0.110	>909.09
*trans*-Resveratrol	17.41	29.37	0.59
Selegiline	67.25	0.020	3362.5
Iproniazid	6.56	7.54	0.87

Each IC_50_ value is the mean ± S.E.M. from five experiments (*n* = 5). * S.I.: *h*MAO-B selectivity index = IC_50_ (*h*MAO-A)/IC_50_ (*h*MAO-B).

**Table 2:** Experimental and calculated inhibitory activities of 3-phenylcoumarin, *trans*-6-styrylcoumarin and *trans*-resveratrol on MAO-A.

Compounds	pIC_50_ MAO-A				
	Exp.	CoMFA	Res	CoMSIA	Res
3-Phenylcoumarin	<4.000	-	-	-	-
*trans*-6-Styrylcoumarin	<4.000	-	-	-	-
*trans*-Resveratrol	4.759	4.767	0.008	4.759	−0.008

**Table 3:** Experimental and calculated inhibitory activities of 3-phenylcoumarin, *trans*-6-styrylcoumarin and *trans*-resveratrol on MAO-B.

Compounds	pIC_50_ MAO-B				
	Exp.	CoMFA	Res	CoMSIA	Res
3-Phenylcoumarin	4.928	5.309	−0.381	5.700	−0.772
*trans*-6-Styrylcoumarin	6.959	7.212	−0.254	7.000	−0.041
*trans*-Resveratrol	4.532	5.173	−0.641	5.362	−0.830

**Table 4:** MM-PBSA binding free energies of MAO-B with 3-phenylcoumarin, 3-(3′-bromophenyl)-6-methylcoumarin, *trans*-6-styrylcoumarin and *trans*-resveratrol between 80ns and 100 ns.

Parameters (Energy)	Enzyme-Ligand Complexes			
	MAO-B and 3-phenylcoumarin (kJ/mol)	MAO-B and 3-(3′-bromophenyl)-6-Methylcoumarin (kJ/mol)	MAO-B and *trans*-6-styrylcoumarin (kJ/mol)	MAO-B and *trans*-resveratrol (kJ/mol)
*Van der Waals*	−145.335 ± 6.596	−137.996 ± 8.218	−152.618 ± 9.322	−178.535 ± 8.654
Electrostatic	−2.253 ± 4.536	−4.754 ± 7.797	−31.762 ± 4.914	0.826 ± 4.532
Polar solvation	99.294 ± 7.536	133.773 ± 19.676	137.238 ± 8.533	94.308 ± 7.084
SASA	−13.845 ± 0.623	−17.202 ± 0.826	−14.723 ± 0.709	−15.502 ± 0.767
Binding free	**−62.139 ± 9.680**	**−26.179 ± 16.851**	**−61.865 ± 10.412**	**−98.903 ± 9.527**

## Data Availability

Data is contained within the article or Supplementary Material.

## Supplementary Materials

The following are available online, Figure S1: The molecular structure of 3-phenylcoumarin with the atom-numbering scheme. Displacement ellipsoids are drawn at the 50% probability level. Figure S2: A view of the title compound. Displacement ellipsoids are drawn at the 50% level. Figure S3: Packing diagram of the title structure. Table S1: Crystal data and structure refinement for *trans*-6-styrylcoumarin. Table S2: Atomic coordinates (×10^4^) and equivalent isotropic displacement parameters (Å^2^ × 10^3^) for *trans-*6-styrylcoumarin. U(eq) is defined as one third of the trace of the orthogonalized Uij tensor. Table S3: Bond lengths [Å] and angles [°] for *trans-*6-styrylcoumarin. Table S4: Anisotropic displacement parameters (Å^2^ × 10^3^) for *trans-*6-styrylcoumarin. The anisotropic displacement factor exponent takes the form: −2π^2^[ h^2^a*^2^U^11^ + … + 2 h k a* b* U^12^]. Table S5: Hydrogen coordinates (×10^4^) and isotropic displacement parameters (Å^2^ × 10^3^). Table S6: Torsion angles [°]. Figure S4: Histogram of frequency distribution data. Figure S5: The superimposed structures of all compounds used in the 3D-QSAR model. Table S7: Chemical structure of dataset used to develop the 3D-QSAR models. Table S8: Inhibitory activity of coumarin and chromone derivatives against MAO-A and MAO-B. Table S9: Field combination of CoMFA and CoMSIA models of MAO-A inhibitors. Table S10: Field combination of CoMFA and CoMSIA models of MAO-B inhibitors. Table S11: Summary of the best CoMFA and CoMSIA models of MAO-A and MAO-B inhibitors. Table S12: Comparison between experimental and predicted values of the CoMFA and CoMSIA models obtained from MAO-A inhibitors. Table S13: Comparison between experimental and predicted values of the CoMFA and CoMSIA models obtained from MAO-B inhibitors. Table S14: Summary of external validation parameters for CoMSIA. Figure S6: Plots of experimental versus predicted pIC_50_ in MAO-A and MAO-B for CoMFA and CoMSIA models. Figure S7: Contour map of 3D-QSAR results for MAO-A inhibitors around the 3-(3′,4′-dihydroxyphenyl)benzo[*f*]coumarin (**84**). Figure S8: Molecular docking of 3-(3′,4′-dihydroxyphenyl)benzo[*f*]coumarin (**84**) on the MAO-A binding pocket. Figure S9: Overlay of *trans*-resveratrol, 3-phenylcoumarin and *trans*-6-styrylcoumarin on the contour maps obtained from the MAO-A CoMFA model. Figure S10: Overlay of *trans*-resveratrol, 3-phenylcoumarin and *trans*-6-styrylcoumarin on the contour maps obtained from the MAO-A CoMSIA model. Figure S11: Schematic enzyme-ligand interactions of 3-phenylcoumarin (A), 3-(3′-bromophenyl)-6-methylcoumarin (B), *trans*-resveratrol (C) and *trans*-6-styrylcoumarin (D) at active site of MAO-B of 100 ns molecular dynamics simulations.

## Sample Availability

Samples of the compounds are available from the authors.
