# Supplementary material for: Coumarin-Resveratrol-Inspired Hybrids as Monoamine Oxidase B Inhibitors: 3-Phenylcoumarin versus trans-6-Styrylcoumarin
Source: Molecules. 2022 Jan 29;27(3):928. doi: 10.3390/molecules27030928 (PMC8838197; doi:10.3390/molecules27030928)
Supplement: Supplementary file 1 [file molecules-27-00928-s001.zip › molecules-1553564-supplementary.pdf]

Supporting Information

Coumarin-resveratrol inspired hybrids as monoamine oxidase B inhibitors: 3-Phenylcoumarin *versus trans*-6-styrylcoumarin

Marco Mellado, César González, Jaime Mella, Luis F. Aguilar, Ismail Celik, Fernanda Borges, Eugenio Uriarte, Giovanna Delogu, Dolores Viña and Maria J. Matos

Table of contents

EXPERIMENTAL SECTION 2

Chemistry 2

General information 2

Synthetic methodology to prepare the 3-phenylcoumarin 2

Synthetic methodology to prepare the *trans*-6-styrylcoumarin 4

X-ray analysis details 7

^{1}H NMR spectra 17

Pharmacology 18

Determination of *in vitro* MAO activity 18

3D-QSAR study 20

Dataset selection and inhibitory activity 20

Molecular Alignment 26

CoMSIA field calculation 27

Internal validation and Partial Least Squares (PLS) analysis 42

External validation 42

Applicability domain calculation 44

Molecular Docking 45

Molecular dynamics simulations 45

ADDITIONAL RESULTS 46

REFERENCES 54

EXPERIMENTAL SECTION

Chemistry

General information. All reagents were purchased from Sigma-Aldrich and used without further purification. All solvents were commercially available grade. All reactions were carried out under argon atmosphere, unless otherwise mentioned. Reaction mixtures were purified by flash column chromatography using Silica Gel high purity grade (Merck grade 9385 pore size 60Å, 230-400 mesh particle size). Reaction mixtures were analyzed by analytical thin-layer chromatography (TLC) using plates precoated with silica gel (Merck 60 F254, 0.25 mm). Visualization was accomplished with UV light (254 nm). ^{1}H NMR and ^{13}C NMR spectra were recorded on a Bruker AMX spectrometer at 250 and 75.47 MHz in the stated solvents ($CDCl_3$ or DMSO-d_6) using tetramethylsilane (TMS) as an internal standard. Chemical shifts were reported in parts per million (ppm) on the δ scale from an internal standard (NMR descriptions: s, singlet; d, doublet; m, multiplet). Mass spectroscopy was performed using a Hewlett-Packard 5988A spectrometer. This system is an automated service utilizing electron impact (EI) ionization. Elemental analyses were performed using a Perkin-Elmer 240B microanalyzer and were within (0.4% of calculated values in all cases).

Synthetic methodology to prepare the 3-phenylcoumarin

3-Phenylcoumarin was prepared according to the protocol described by Matos *et al.* [1]. A solution of 2-hydroxybenzaldehyde (7.37 mmol, 0.9 g) and the phenylacetic acid (9.21 mmol, 1.25 g) in DMSO (15 mL) was prepared. DCC (11.50 mmol, 2.37 g) was added, and the mixture was heated at 110 °C for 24 hours. Ice (100 mL) and acetic acid (10 mL) were added to the reaction mixture. After keeping it at room temperature for 2 hours, the mixture was extracted with ether (3 x 25 mL). The organic layer was extracted with sodium bicarbonate solution (50 ml, 5%) and then water (20 mL). The solvent was evaporated under vacuum, and the dry residue was purified by flash chromatography (hexane/ethyl acetate 9:1). A white solid was obtained in a yield of 67% (1.1 g). Suitable crystals for X-ray studies were grown from slow evaporation from acetone/ethanol [2]. The X-ray analysis of this compound is represented in Figure S1.

Mp. 131–132 °C. ^{1}H NMR (300 MHz, $CDCl_3$) δ: 7.34–7.54 (m, 5H, H-6, H-8, H-9, H-11, H-13), 7.56–7.66 (m, 2H, H-10, H-12), 7.72–7.80 (m, 2H, H-5, H-7), 7.90 (s, 1H, H-4).* ^{13}C NMR (75.47 MHz, $CDCl_3$) δ: 116.5, 119.7, 124.5, 127.9, 128.4, 128.5, 128.5, 128.9, 131.4, 134.7, 139.9, 153.5, 160.6.* DEPT: 116.5, 124.5, 127.9, 128.4, 128.5, 128.5, 131.4, 139.9. MS *m/z*: 222 (M^+, 100). Anal. Calcd for $C_{15}H_{10}O_2$: C, 81.07; H, 4.54. Found: C, 81.02; H, 4.52.

* numbers according Figure S1.

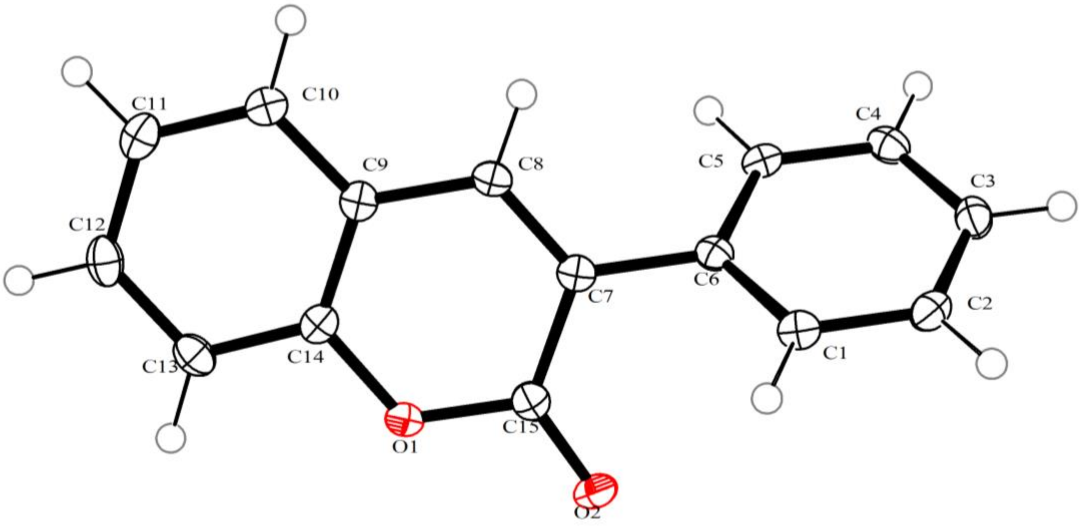

Figure S1. The molecular structure of 3-phenylcoumarin with the atom-numbering scheme. Displacement ellipsoids are drawn at the 50% probability level.

Synthetic methodology to prepare the *trans*-6-styrylcoumarin

1. General procedure for the preparation of the 6-bromomethylcoumarin. To a solution of 6-methylcoumarin (6.24 mmol, 1.0 g) in carbon tetrachloride (CCl_4, 22.6 mL), *N*-bromosuccinimide (NBS, 7.49 mmol, 1.33 g) and a catalytic amount of azobisisobutyronitrile (AIBN) were added. The reaction mixture was refluxed until the disappearance of the starting material (about 24 h). The succinimide was rapidly filtered off and the desired solid product recovered after cooling.

Mp. 171-172 °C. ^1H NMR (250 MHz, $CDCl_3$) δ: 4.54 (s, 2H, CH_2), 6.44 (d, 1H, H-3, *J*=9.6), 7.31 (d, 1H, H-8, *J*=8.4), 7.54 (dd, 1H, H-7, *J*=8.4, *J*=2.2), 7.57 (d, 1H, H-5, *J*=2.2), 7.70 (d, 1H, H-4, *J*=9.6). ^{13}C NMR (75.47 MHz, $CDCl_3$) δ: 32.3, 117.7, 117.9, 118.9, 128.6, 132.9, 134.7, 143.3, 153.9, 160.9. DEPT: 32.3, 117.7, 117.9, 128.6, 132.9, 143.3. MS *m/z* (%): 238 (M^+, 5), 159 (100). Anal. Calcd for $C_{10}H_{17}BrO_2$: C, 50.24; H, 2.95. Found: C, 50.18; H, 2.87.

2. General procedure for the preparation of the 6-methylcoumarintriphenylphosphonium bromide. To a solution of 6-bromomethylcoumarin (12.55 mmol, 3.0 g) in toluene (26.79 mL), triphenylphosphine (16.31 mmol, 4.28 g) was added. The solution was heated at reflux for 12 h under argon. The resulting precipitate was collected and recrystallized from ethanol as colorless crystals.

Mp. 335-336 °C. ^1H NMR (250 MHz, $CDCl_3$) δ: 5.57 (d, 2H, CH_2, *J*=14.6), 6.09 (d, 1H, H-3, *J*=9.5), 6.73 (d, 1H, H-8, *J*=8.6), 7.09 (m, 1H, H-7), 7.29 (d, 1H, H-4, *J*=9.5), 7.37-7.44 (m, 7H), 7.52-7.64 (m, 9H). ^{13}C NMR (75.47 MHz, $CDCl_3$) δ: 34.4, 117.2, 117.3, 118.5, 128.6, 132.9, 134.7, 143.3, 153.9, 160.9. IR (KBr) υm (cm^{-1}): 2992, 2880, 2885, 2784, 1725, 1107. MS *m/z* (%):421 (M^+, 100). Anal. Calcd for $C_{28}H_{22}BrO_2P$: C, 67.08; H, 4.42. Found: C, 67.01; H, 4.44.

3. Wittig reaction to prepare the *trans*-6-styrylcoumarin. *trans*-6-Styrylcoumarin was prepared according to the protocol described by Cushman *et al.* [3]. NaH (1.5 mmol, 20 mg) was added in portions to a well-stirred suspension of 6-methylcoumarintriphenylphosphonium bromide (1.0 mmol, 500 mg) and benzaldehyde (1.0 mmol, 106 mg) in THF (20 mL) under an argon atmosphere at 0-5 ºC, and the mixture was

allowed to warm to room temperature. After an additional stirring for 16 hours, excess NaH was quenched by the addition of methanol (1 mL). Solvents from the reaction mixture were evaporated at reduced pressure, and the residue was purified by flash chromatography followed by preparative thin layer chromatography using 5% EtOH in hexane as the eluent. *trans*-6-Styrylcoumarin and *cis*-6-styrylcoumarin were obtained as solids. *Cis* derivative was obtained in a small amount, impossible to be characterized. Suitable crystals of the *trans* isomer for X-ray studies were grown from slow evaporation from acetone/ethanol.

Mp. 134–135 °C. ^{1}H NMR (250 MHz, $CDCl_3$) δ: 6.46 (d, 1H, H-3, *J*=9.5), 7.10 (s, 2H), 7.30–7.33 (m, 2H), 7.35 (d, 1H, H-11, *J*=15.5), 7.40 (d, 1H, H-12, *J*=15.5), 7.53 (d, 2H, *J*=7.4), 7.58 (d, 1H, H-6, *J*=2.0), 7.70 (dd, 1H, H-8, *J*=6.7, 2.0), 7.74 (d, 1H, H-4, *J*=9.5).* ^{13}C NMR (75.47 MHz, $CDCl_3$) δ: 117.0 (C3), 117.2 (C9), 119.0 (C5), 125.4 (C6), 126.5 (C11, C12), 126.6 (C14, C18), 128.0 (C15), 128.8 (C17), 129.7 (C16), 129.8 (C8), 134.0 (C7), 136.7 (C13), 143.3 (C4), 153.4 (C10), 160.6 (C=O).* DEPT: 117.0, 117.2, 125.4, 126.5, 126.6, 128.0, 128.8, 129.7, 136.7. MS *m/z*: 248 (M^+, 100). Anal. Calcd for $C_{17}H_{12}O_2$: C, 82.24; H, 4.87. Found: C, 82.27; H, 4.90.

* numbers according Figure S2.

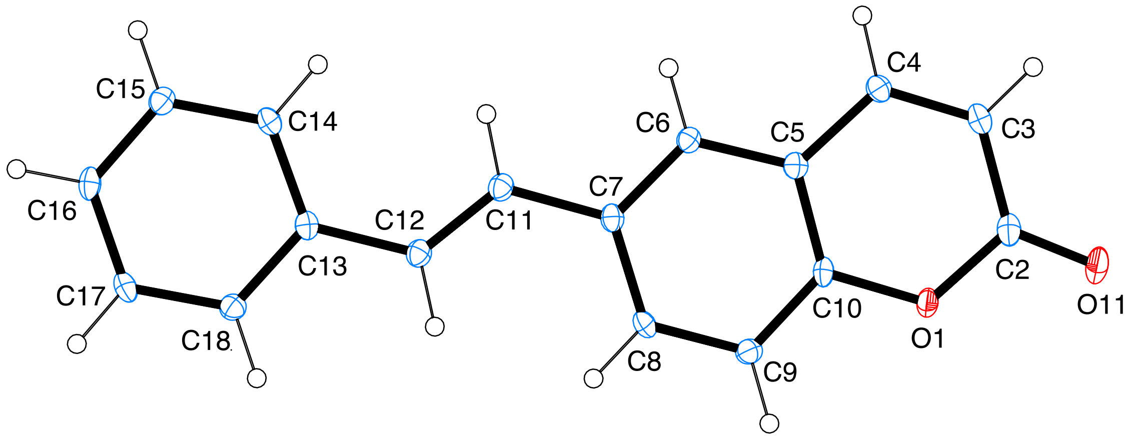

Figure S2. A view of the title compound. Displacement ellipsoids are drawn at the 50% level.

The X-ray analysis of this compound (Figure S2) aims to contribute to the elucidation of structural requirements needed to understand the geometry of the most abundant stereoisomer, the *trans*-6-styrylcoumarin. Packing diagram of the structure allows the interpretation of the spatial orientation of the molecules (Figure S3).

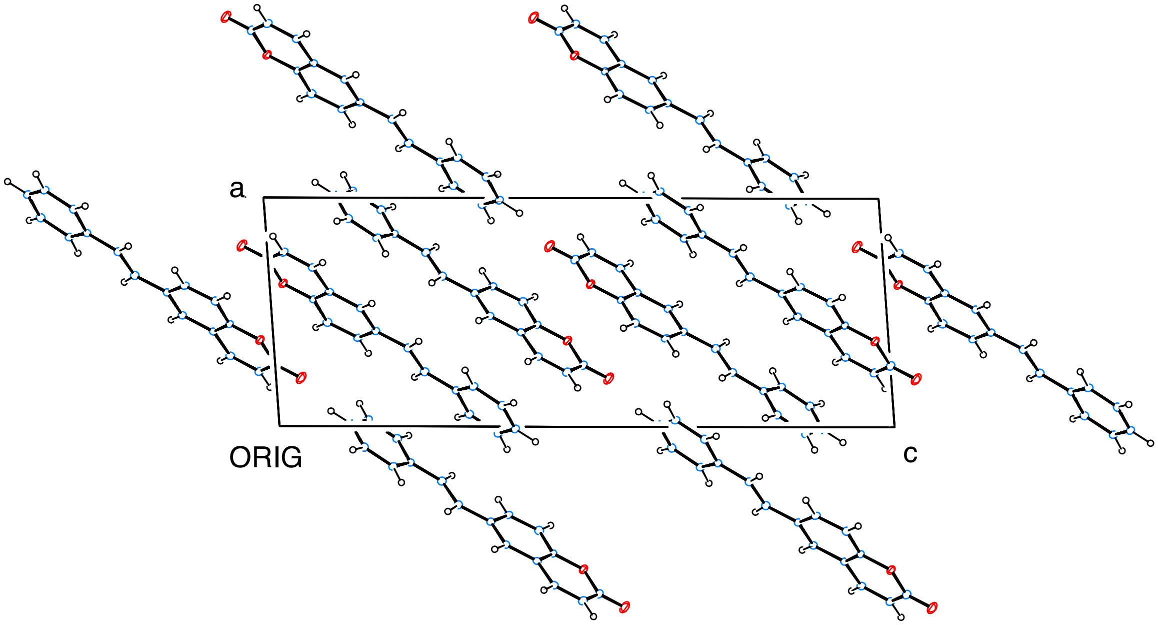

Figure S3. Packing diagram of the title structure.

X-ray analysis details

Table S1. Crystal data and structure refinement for *trans*-6-styrylcoumarin.

Identification code	*trans*-6-styrylcoumarin	
Empirical formula	$C_{17}H_{12}O_2$	
Formula weight	248.27	
Temperature	100(2) K	
Wavelength	0.71073 Å	
Crystal system	Monoclinic	
Space group	$P2_1/c$	
Unit cell dimensions	a = 8.9047(5) Å	α = 90°
	b = 5.7602(3) Å	β = 94.138(3)°
	c = 23.9481(11) Å	γ = 90°
Volume	1225.16(11) Å^3	
Z	4	
Density (calculated)	1.346 Mg/m^3	
Absorption coefficient	0.087 mm^{-1}	
F(000)	520	
Crystal size	0.70 x 0.15 x 0.03 mm^3	
Theta range for data collection	2.29 to 26.39°	
Index ranges	-11<=h<=11, 0<=k<=7, 0<=l<=29	
Reflections collected	17766	
Independent reflections	2488 [R(int) = 0.0443]	
Completeness to theta = 26.39°	99.2 %	
Absorption correction	Semi-empirical from equivalents	
Max. and min. transmission	0.9773 and 0.8720	
Refinement method	Full-matrix least-squares on F^2	
Data / restraints / parameters	2488 / 0 / 172	
Goodness-of-fit on F^2	1.032	
Final R indices [I>2sigma(I)]	R1 = 0.0449, wR2 = 0.1034	
R indices (all data)	R1 = 0.0734, wR2 = 0.1175	
Largest diff. peak and hole	0.182 and -0.233 e.Å^{-3}	

Data collection: *APEX2* v2009-3.0 (BRUKER AXS, Madison); cell refinement: *APEX2* v2009-3.0 (BRUKER AXS, Madison); data reduction: *APEX2* v2009-3.0 (BRUKER AXS,

Madison); program(s) used to solve structure: *SIR97* [4]; program(s) used to refine structure: *SHELXL97* [5]; molecular graphics: *ORTEP-3 for Windows* [6]; software used to prepare material for publication: *WinGX* publication routines [7].

Special details

Geometry. All s.u.'s (except the s.u. in the dihedral angle between two l.s. planes) are estimated using the full covariance matrix. The cell s.u.'s are considered individually in the estimation of s.u.'s in distances, angles and torsion angles; correlations between s.u.'s in cell parameters are only used when they are defined by crystal symmetry. An approximate (isotropic) treatment of cell s.u.'s is used for estimating s.u.'s involving l.s. planes.

Refinement. Refinement of F^2 against ALL reflections. The weighted *R*-factor *wR* and goodness of fit *S* are based on F^2, conventional *R*-factors *R* are based on *F*, with *F* set to zero for negative F^2. The threshold expression of $F^2 > 2\sigma(F^2)$ is used only for calculating *R*-factors(gt) *etc*. and is not relevant to the choice of reflections for refinement. *R*-factors based on F^2 are statistically about twice as large as those based on *F*, and *R*- factors based on ALL data will be even larger.

Table S2. Atomic coordinates ($x10^4$) and equivalent isotropic displacement parameters ($Å^2 x10^3$) for *trans*-6-styrylcoumarin. U(eq) is defined as one third of the trace of the orthogonalized U^{ij} tensor.

	x	y	z	U(eq)
C(2)	2689(2)	3977(3)	5005(1)	23(1)
C(3)	2390(2)	6204(3)	4737(1)	22(1)
C(4)	3066(2)	6839(3)	4280(1)	19(1)
C(5)	4125(2)	5312(3)	4035(1)	17(1)
C(6)	4836(2)	5817(3)	3546(1)	17(1)
C(7)	5835(2)	4263(3)	3327(1)	18(1)
C(8)	6128(2)	2166(3)	3618(1)	19(1)
C(9)	5457(2)	1639(3)	4103(1)	19(1)
C(10)	4451(2)	3210(3)	4305(1)	18(1)
C(11)	6571(2)	4881(3)	2817(1)	20(1)
C(12)	7610(2)	3618(3)	2578(1)	19(1)
C(13)	8421(2)	4232(3)	2087(1)	18(1)
C(14)	8245(2)	6347(3)	1802(1)	19(1)
C(15)	9085(2)	6840(3)	1352(1)	20(1)
C(16)	10103(2)	5223(3)	1169(1)	21(1)
C(17)	10295(2)	3121(3)	1447(1)	21(1)
C(18)	9461(2)	2644(3)	1900(1)	20(1)
O(1)	3763(1)	2581(2)	4780(1)	21(1)
O(11)	2108(1)	3211(2)	5406(1)	33(1)

Table S3. Bond lengths [Å] and angles [°] for *trans*-6-styrylcoumarin.

C(2)-O(11)	1.2065(19)
C(2)-O(1)	1.3873(19)
C(2)-C(3)	1.450(2)
C(3)-C(4)	1.338(2)
C(3)-H(3)	0.9500
C(4)-C(5)	1.444(2)
C(4)-H(4)	0.9500
C(5)-C(10)	1.393(2)
C(5)-C(6)	1.403(2)
C(6)-C(7)	1.391(2)
C(6)-H(6)	0.9500
C(7)-C(8)	1.409(2)
C(7)-C(11)	1.471(2)
C(8)-C(9)	1.379(2)
C(8)-H(8)	0.9500
C(9)-C(10)	1.384(2)
C(9)-H(9)	0.9500
C(10)-O(1)	1.3802(17)
C(11)-C(12)	1.338(2)
C(11)-H(11)	0.9500
C(12)-C(13)	1.467(2)
C(12)-H(12)	0.9500
C(13)-C(18)	1.398(2)
C(13)-C(14)	1.400(2)
C(14)-C(15)	1.386(2)
C(14)-H(14)	0.9500
C(15)-C(16)	1.392(2)
C(15)-H(15)	0.9500
C(16)-C(17)	1.387(2)
C(16)-H(16)	0.9500
C(17)-C(18)	1.386(2)
C(17)-H(17)	0.9500
C(18)-H(18)	0.9500

O(11)-C(2)-O(1)	116.32(16)
O(11)-C(2)-C(3)	126.86(16)
O(1)-C(2)-C(3)	116.82(14)
C(4)-C(3)-C(2)	121.65(15)
C(4)-C(3)-H(3)	119.2
C(2)-C(3)-H(3)	119.2
C(3)-C(4)-C(5)	120.71(16)
C(3)-C(4)-H(4)	119.6
C(5)-C(4)-H(4)	119.6
C(10)-C(5)-C(6)	118.40(14)
C(10)-C(5)-C(4)	117.55(14)
C(6)-C(5)-C(4)	124.06(15)
C(7)-C(6)-C(5)	121.39(15)
C(7)-C(6)-H(6)	119.3
C(5)-C(6)-H(6)	119.3
C(6)-C(7)-C(8)	117.84(14)
C(6)-C(7)-C(11)	119.48(15)
C(8)-C(7)-C(11)	122.66(14)
C(9)-C(8)-C(7)	121.93(15)
C(9)-C(8)-H(8)	119.0
C(7)-C(8)-H(8)	119.0
C(8)-C(9)-C(10)	118.73(15)
C(8)-C(9)-H(9)	120.6
C(10)-C(9)-H(9)	120.6
O(1)-C(10)-C(9)	116.94(14)
O(1)-C(10)-C(5)	121.34(14)
C(9)-C(10)-C(5)	121.71(15)
C(12)-C(11)-C(7)	125.92(16)
C(12)-C(11)-H(11)	117.0
C(7)-C(11)-H(11)	117.0
C(11)-C(12)-C(13)	127.39(16)
C(11)-C(12)-H(12)	116.3
C(13)-C(12)-H(12)	116.3
C(18)-C(13)-C(14)	117.83(14)
C(18)-C(13)-C(12)	118.35(15)
C(14)-C(13)-C(12)	123.80(15)

C(15)-C(14)-C(13)	120.67(15)
C(15)-C(14)-H(14)	119.7
C(13)-C(14)-H(14)	119.7
C(14)-C(15)-C(16)	120.52(16)
C(14)-C(15)-H(15)	119.7
C(16)-C(15)-H(15)	119.7
C(17)-C(16)-C(15)	119.60(15)
C(17)-C(16)-H(16)	120.2
C(15)-C(16)-H(16)	120.2
C(18)-C(17)-C(16)	119.64(15)
C(18)-C(17)-H(17)	120.2
C(16)-C(17)-H(17)	120.2
C(17)-C(18)-C(13)	121.73(15)
C(17)-C(18)-H(18)	119.1
C(13)-C(18)-H(18)	119.1
C(10)-O(1)-C(2)	121.81(13)

Table S4. Anisotropic displacement parameters ($Å^2 x 10^3$) for *trans*-6-styrylcoumarin. The anisotropic displacement factor exponent takes the form: $-2\pi^2[h^2 a^{*2} U^{11} + ... + 2 h k a^* b^* U^{12}]$.

	U^{11}	U^{22}	U^{33}	U^{23}	U^{13}	U^{12}
C(2)	19(1)	31(1)	19(1)	0(1)	5(1)	-1(1)
C(3)	17(1)	26(1)	22(1)	-4(1)	5(1)	1(1)
C(4)	15(1)	21(1)	21(1)	-1(1)	1(1)	0(1)
C(5)	13(1)	20(1)	18(1)	-1(1)	-1(1)	-2(1)
C(6)	15(1)	19(1)	18(1)	1(1)	1(1)	-2(1)
C(7)	14(1)	22(1)	18(1)	-1(1)	1(1)	-2(1)
C(8)	14(1)	22(1)	22(1)	-2(1)	4(1)	2(1)
C(9)	18(1)	18(1)	21(1)	2(1)	1(1)	0(1)
C(10)	13(1)	26(1)	14(1)	0(1)	3(1)	-3(1)
C(11)	17(1)	22(1)	20(1)	2(1)	2(1)	-1(1)
C(12)	17(1)	21(1)	20(1)	1(1)	2(1)	-2(1)
C(13)	14(1)	23(1)	16(1)	-2(1)	1(1)	-2(1)
C(14)	15(1)	22(1)	21(1)	-2(1)	3(1)	1(1)
C(15)	18(1)	23(1)	20(1)	2(1)	1(1)	-1(1)
C(16)	15(1)	30(1)	18(1)	0(1)	4(1)	-5(1)
C(17)	14(1)	27(1)	20(1)	-4(1)	4(1)	3(1)
C(18)	17(1)	21(1)	20(1)	2(1)	0(1)	0(1)
O(1)	19(1)	26(1)	19(1)	4(1)	7(1)	1(1)
O(11)	33(1)	41(1)	27(1)	8(1)	16(1)	0(1)

Table S5. Hydrogen coordinates (x10^4) and isotropic displacement parameters (Å^{2}x10^3).

	x	y	z	U(eq)
H(3)	1698	7238	4890	26
H(4)	2846	8310	4114	23
H(6)	4631	7251	3360	20
H(8)	6808	1083	3475	23
H(9)	5681	225	4296	23
H(11)	6281	6306	2641	24
H(12)	7851	2157	2746	23
H(14)	7542	7455	1919	23
H(15)	8966	8293	1166	24
H(16)	10663	5558	856	25
H(17)	10993	2013	1327	25
H(18)	9600	1201	2089	23

Table S6. Torsion angles [°].

O(11)-C(2)-C(3)-C(4)	-177.50(16)
O(1)-C(2)-C(3)-C(4)	3.1(2)
C(2)-C(3)-C(4)-C(5)	0.0(2)
C(3)-C(4)-C(5)-C(10)	-2.3(2)
C(3)-C(4)-C(5)-C(6)	177.61(15)
C(10)-C(5)-C(6)-C(7)	0.6(2)
C(4)-C(5)-C(6)-C(7)	-179.31(14)
C(5)-C(6)-C(7)-C(8)	-0.8(2)
C(5)-C(6)-C(7)-C(11)	-179.15(13)
C(6)-C(7)-C(8)-C(9)	0.0(2)
C(11)-C(7)-C(8)-C(9)	178.33(14)
C(7)-C(8)-C(9)-C(10)	0.9(2)
C(8)-C(9)-C(10)-O(1)	177.76(13)
C(8)-C(9)-C(10)-C(5)	-1.2(2)
C(6)-C(5)-C(10)-O(1)	-178.45(12)
C(4)-C(5)-C(10)-O(1)	1.4(2)
C(6)-C(5)-C(10)-C(9)	0.5(2)
C(4)-C(5)-C(10)-C(9)	-179.67(14)
C(6)-C(7)-C(11)-C(12)	176.21(15)
C(8)-C(7)-C(11)-C(12)	-2.1(2)
C(7)-C(11)-C(12)-C(13)	-176.67(15)
C(11)-C(12)-C(13)-C(18)	-179.38(15)
C(11)-C(12)-C(13)-C(14)	2.4(3)
C(18)-C(13)-C(14)-C(15)	-0.4(2)
C(12)-C(13)-C(14)-C(15)	177.88(14)
C(13)-C(14)-C(15)-C(16)	1.1(2)
C(14)-C(15)-C(16)-C(17)	-1.2(2)
C(15)-C(16)-C(17)-C(18)	0.6(2)
C(16)-C(17)-C(18)-C(13)	0.1(2)
C(14)-C(13)-C(18)-C(17)	-0.2(2)
C(12)-C(13)-C(18)-C(17)	-178.56(14)
C(9)-C(10)-O(1)-C(2)	-177.20(14)
C(5)-C(10)-O(1)-C(2)	1.8(2)
O(11)-C(2)-O(1)-C(10)	176.58(14)

C(3)-C(2)-O(1)-C(10)	-3.9(2)

^{1}H NMR spectra

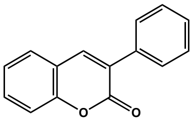

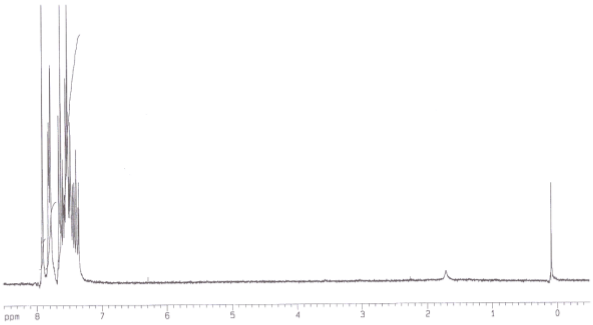

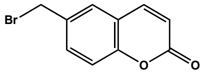

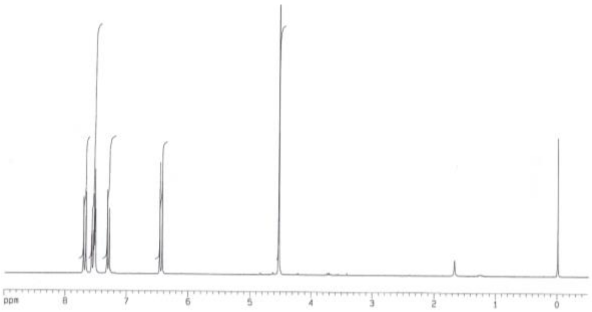

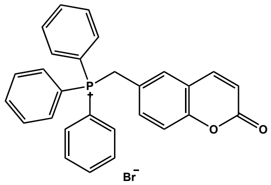

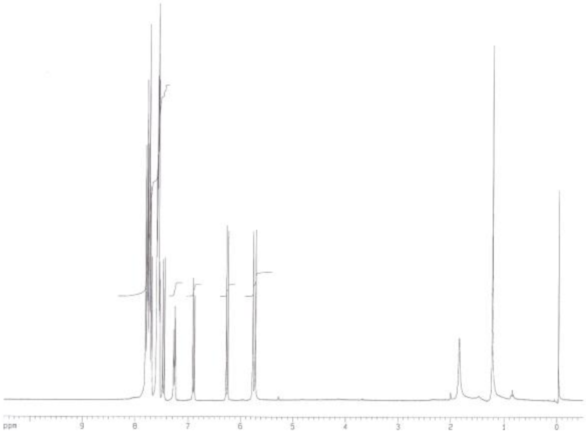

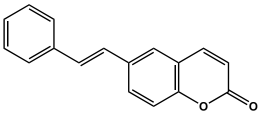

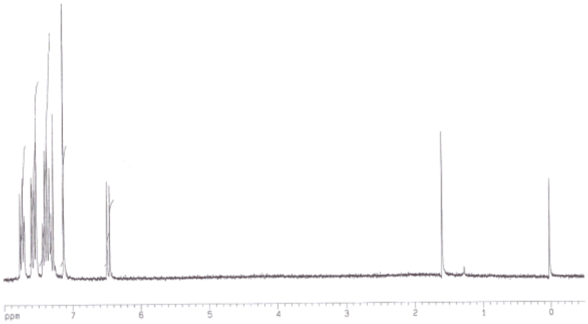

Pharmacology

Determination of *in vitro* MAO activity

The effects of the coumarin-resveratrol derivatives and *trans*-resveratrol on *h*MAO enzymatic activity were evaluated by a fluorometric method following the experimental protocol previously described by us. Briefly, 50 µL of sodium phosphate buffer (0.05 M, pH 7.4) containing the test molecules (compounds or reference inhibitors) in different concentrations and adequate amounts of recombinant *h*MAO-A or *h*MAO-B [adjusted to obtain in our experimental conditions the same reaction velocity (*h*MAO-A: 1.1 µg protein; specific activity: 150 nmol of *p*-tyramine oxidized to *p*-hydroxyphenylacetaldehyde/min/mg protein; *h*MAO-B: 7.5 µg protein; specific activity: 22 nmol of *p*-tyramine transformed/ min/mg protein)] were incubated for 10 min at 37 °C in a flat-black bottom 96-well microtest plate, placed in the dark fluorimeter chamber. After this incubation period, the reaction was started by adding 50 µL of the mixture containing (final concentrations) 200 µM of the Amplex® Red reagent, 1 U/mL of horseradish peroxidase and 1 mM of *p*-tyramine. The production of H_2O_2 and, consequently, of resorufin, was quantified at 37 °C in a multidetection microplate fluorescence reader (Fluo-star OptimaTM, BMG LABTECH, Offenburg, Germany) based on the fluorescence generated (excitation, 545 nm, emission, 590 nm) over a 10 min period, in which the fluorescence increased linearly. Control experiments were carried out simultaneously by replacing the tested molecules with appropriate dilutions of the vehicles. In addition, the possible capacity of these molecules to modify the fluorescence generated in the reaction mixture, due to non-enzymatic inhibition (i.e. for directly reacting with Amplex® Red reagent), was determined by adding these molecules to solutions containing only the Amplex® Red reagent in sodium phosphate buffer. The specific fluorescence emission (used to obtain the final results) was calculated after subtraction of the background activity, which was determined from wells containing all components except the *h*MAO isoforms, which were replaced by sodium phosphate buffer solution.

MAO activity of the test compounds and reference inhibitors is expressed as IC_{50} values, that is, the concentration of each drug required to produce a 50% decrease on the control value

activity of the MAO isoforms. The corresponding IC_{50} values were calculated by using the Origin 5.0 software (Microcal Software Inc., Northampton, MA, USA), from the equations of the lines obtained by linear regression (methods least squares) of the resulting points to represent the log of the molar concentration of the test compound (x axis) *versus* the percentage inhibition of the control MAO activity achieved with corresponding concentrations of each compound (y axis). This linear regression was performed by using data obtained with 4–6 concentrations of each test compound capable of inhibiting the control enzyme activity of the MAO isoenzymes by between 20 and 80%. Also, the [IC_{50}(MAO-A)]/[IC_{50}(MAO-B)] ratio was calculated as an indicator of the rate of selectivity in the inhibition of both isoforms.

3D-QSAR study

Dataset selection and inhibitory activity

3D-QSAR studies [8-12] were performed on a set of 30 compounds (MAO-A inhibitors) and 157 compounds (MAO-B inhibitors), and the structures of all the compounds are represented in Table S7 [9,13-29]. The derivatives displayed MAO-A and MAO-B inhibitory activities, as represented in Table S8. The IC_{50} values were converted to pIC_{50} ($-\log IC_{50}$). The compounds were randomly divided into training (107 compounds, 71%) and test (43 compounds, 29%) sets. The distribution of pIC_{50} values for the whole set, the training set and the test set, is shown in Figure S4.

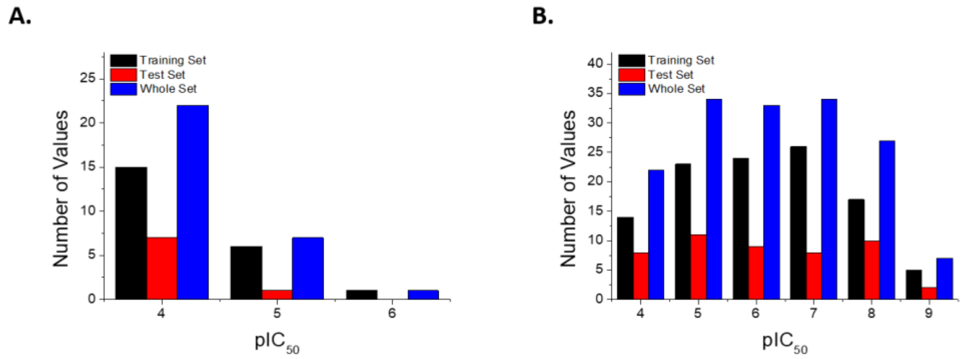

Figure S4. Histogram of frequency distribution data. **A.** Distribution of compounds on MAO-A. **B.** Distribution of compounds on MAO-B.

Table S7. Chemical structure of dataset used to develop the 3D-QSAR models.

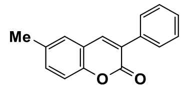 **1 [13]**	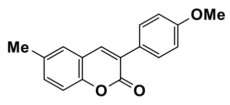 **2 [13]**	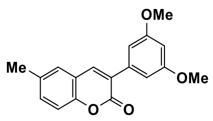 **3 [13]**	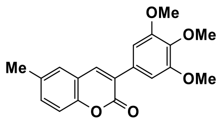 **4 [13]**
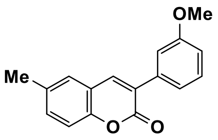	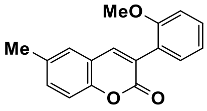 **6 [14]**	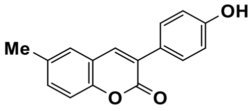 **7 [14]**	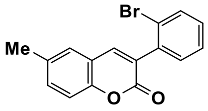 **8 [14]**

**5 [14]**			
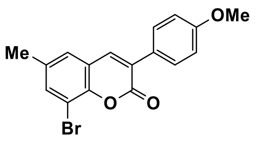 **9 [14]**	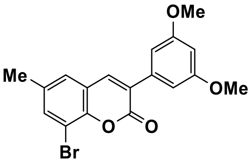 **10 [14]**	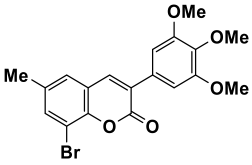 **11 [14]**	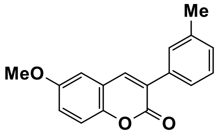 **12 [15]**
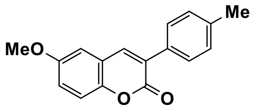 **13 [15]**	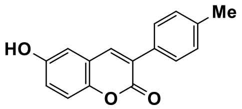 **14 [15]**	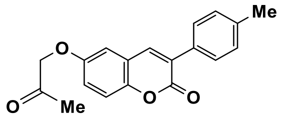 **15 [15]**	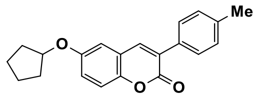 **16 [15]**
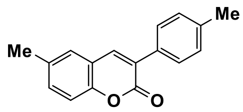 **17 [15]**	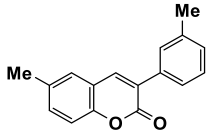 **18 [15]**	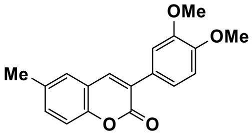 **19 [15]**	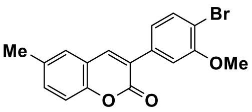 **20 [15]**
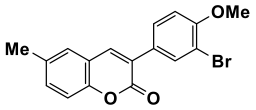 **21 [15]**	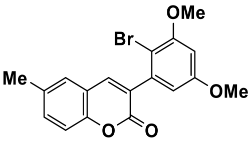 **22 [15]**	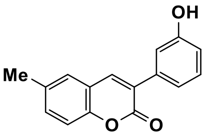 **23 [15]**	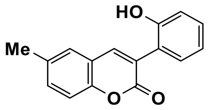 **24 [15]**
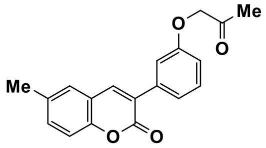 **25 [15]**	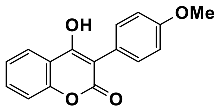 **26 [16]**	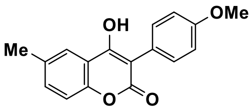 **27 [16]**	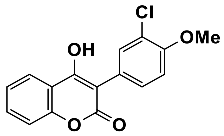 **28 [16]**
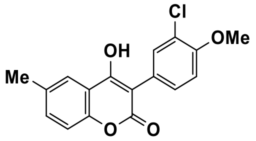 **29 [16]**	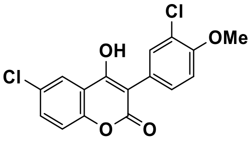 **30 [16]**	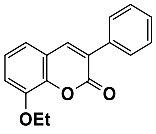 **31 [17]**	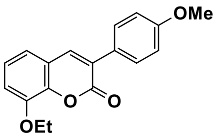 **32 [17]**

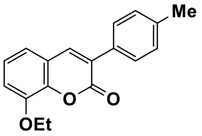 **33 [17]**	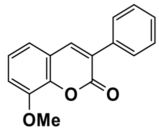 **34 [17]**	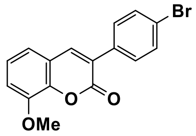 **35 [17]**	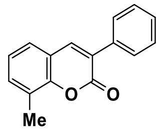 **36 [17]**
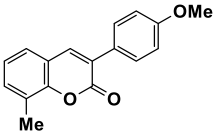 **37 [17]**	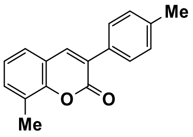 **38 [17]**	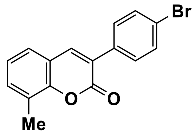 **39 [17]**	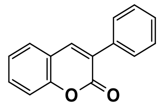 **40 [18]**
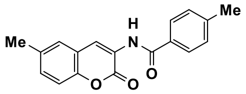 **41 [18]**	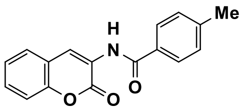 **42 [18]**	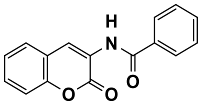 **43 [18]**	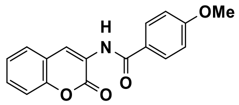 **44 [18]**
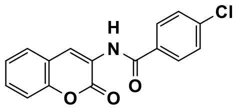 **45 [18]**	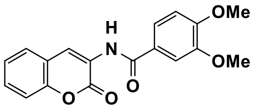 **46 [18]**	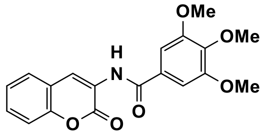 **47 [18]**	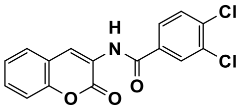 **48 [18]**
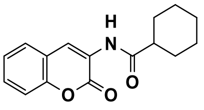 **49 [18]**	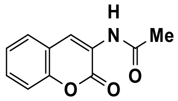 **50 [18]**	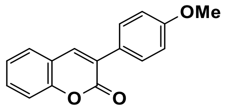 **51 [19]**	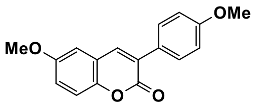 **52 [19]**
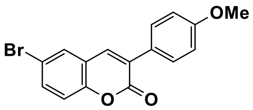 **53 [19]**	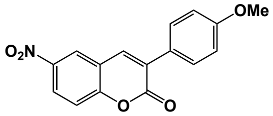 **54 [19]**	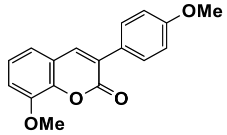 **55 [19]**	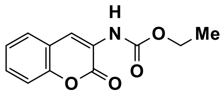 **56 [20]**
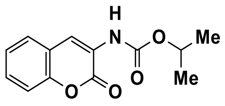 **57 [20]**	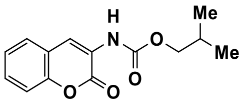 **58 [20]**	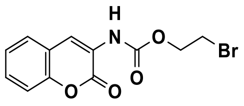 **59 [20]**	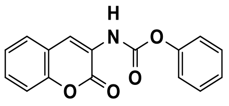 **60 [20]**

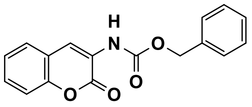

61 [20]

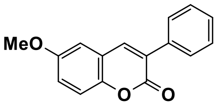

62 [21]

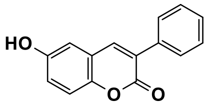

63 [21]

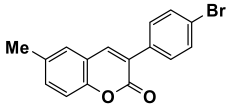

64 [21]

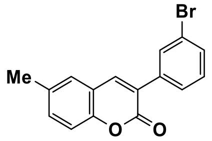

65 [21]

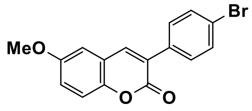

66 [21]

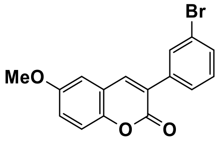

67 [21]

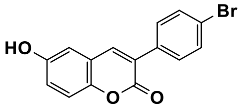

68 [21]

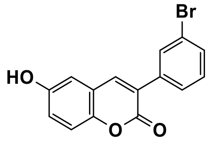

69 [21]

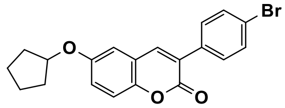

70 [21]

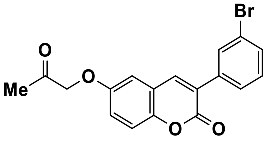

71 [21]

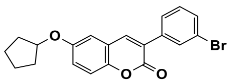

72 [21]

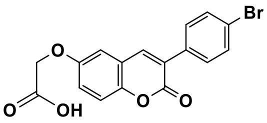

73 [21]

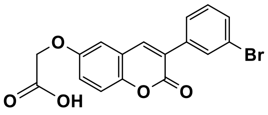

74 [21]

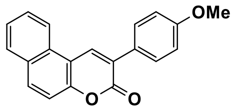

75 [22]

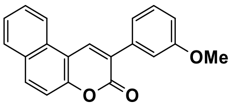

76 [22]

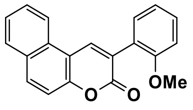

77 [22]

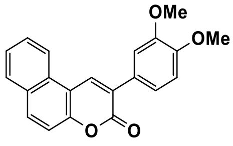

78 [22]

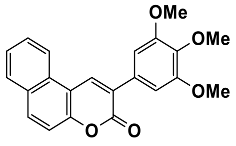

79 [22]

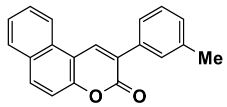

80 [22]

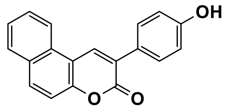

81 [22]

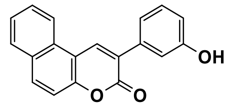

82 [22]

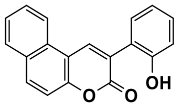

83 [22]

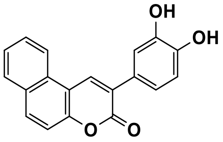

84 [22]

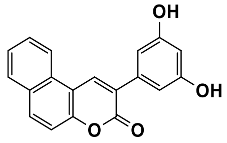

85 [22]

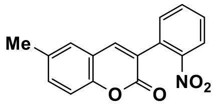

86 [23]

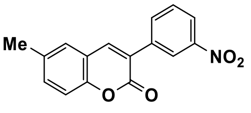

87 [23]

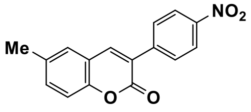

88 [23]

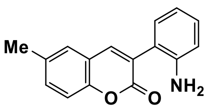 **89 [23]**	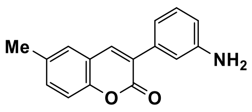 **90 [23]**	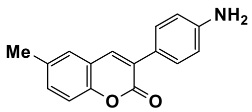 **91 [23]**	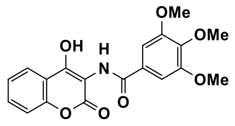 **92 [24]**
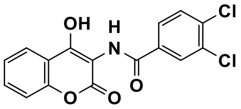 **93 [24]**	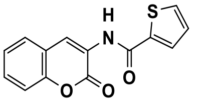 **94 [24]**	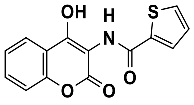 **95 [24]**	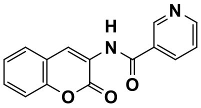 **96 [24]**
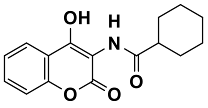 **97 [24]**	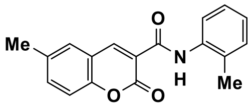 **98 [25]**	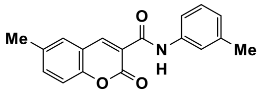 **99 [25]**	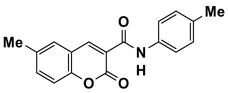 **100 [25]**
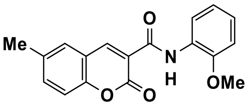 **101 [25]**	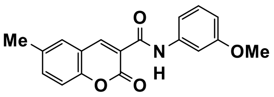 **102 [25]**	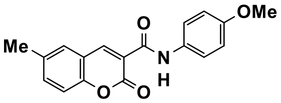 **103 [25]**	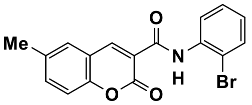 **104 [25]**
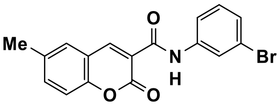 **105 [25]**	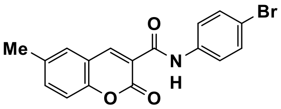 **106 [25]**	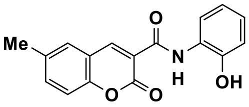 **107 [25]**	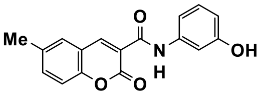 **108 [25]**
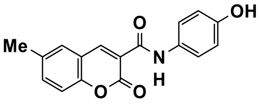 **109 [25]**	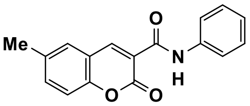 **110 [26]**	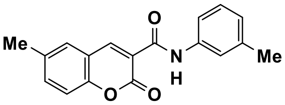 **111 [26]**	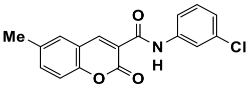 **112 [26]**
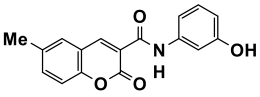 **113 [26]**	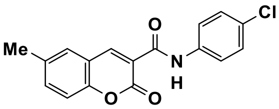 **114 [26]**	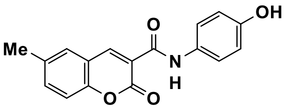 **115 [26]**	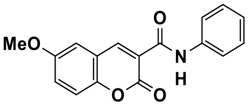 **116 [26]**
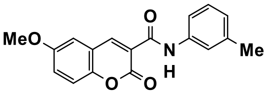 **117 [26]**	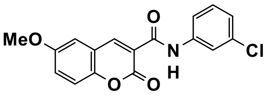	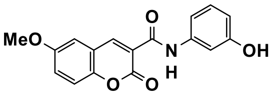 **119 [26]**	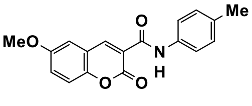 **120 [26]**

	**118 [26]**		
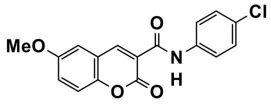<br>**121 [26]**	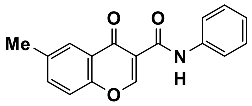<br>**122 [26]**	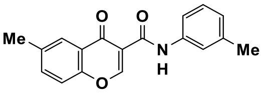<br>**123 [26]**	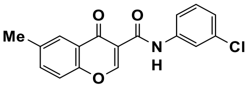<br>**124 [26]**
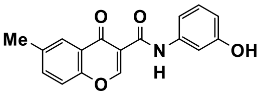<br>**125 [26]**	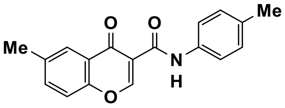<br>**126 [26]**	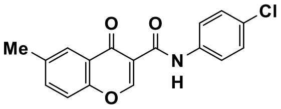<br>**127 [26]**	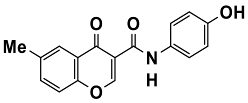<br>**128 [26]**
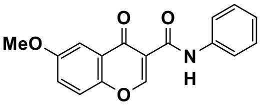<br>**129 [26]**	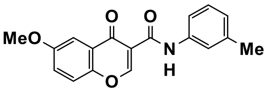<br>**130 [26]**	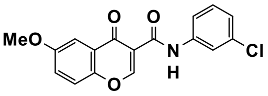<br>**131 [26]**	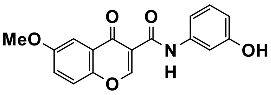<br>**132 [26]**
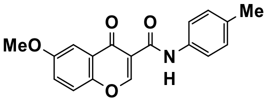<br>**133 [26]**	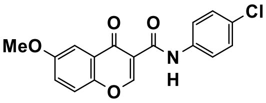<br>**134 [26]**	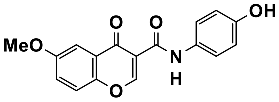<br>**135 [26]**	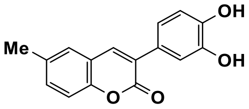<br>**136 [27]**
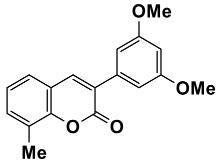<br>**137 [27]**	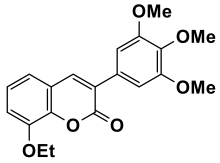<br>**138 [27]**	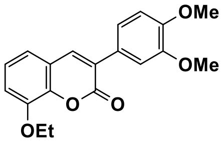<br>**139 [27]**	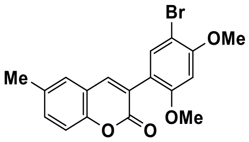<br>**140 [27]**
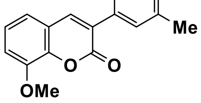<br>**141 [27]**	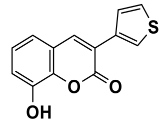<br>**142 [9]**	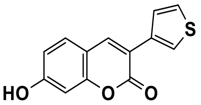<br>**143 [9]**	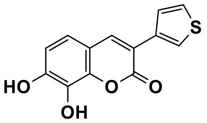<br>**144 [9]**
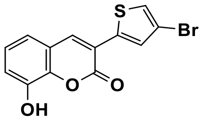<br>**145 [9]**	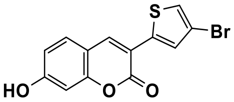<br>**146 [9]**	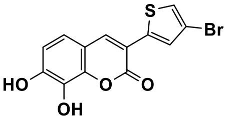<br>**147 [9]**	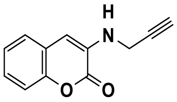<br>**148 [28]**

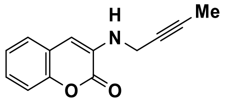 **149 [28]**	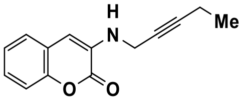 **150 [28]**	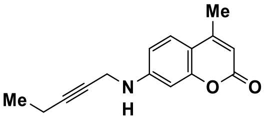 **151 [28]**	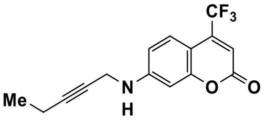 **152 [28]**
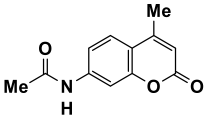 **153 [29]**	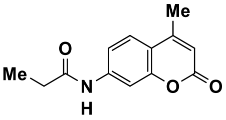 **154 [29]**	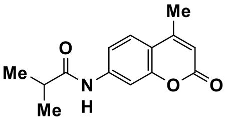 **155 [29]**	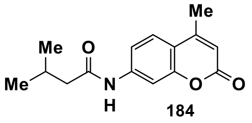 **156 [29]**
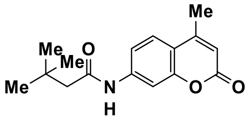 **157 [29]**	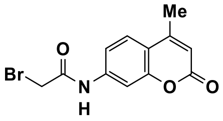 **158 [29]**	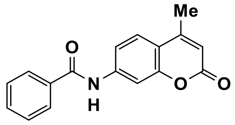 **159 [29]**	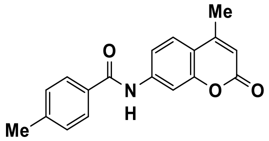 **160 [29]**
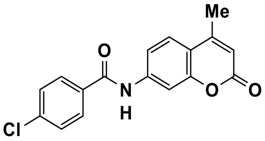 **161 [29]**	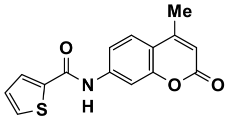 **162 [29]**	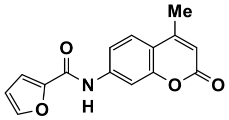 **163 [29]**	

Molecular Alignment

3D-QSAR studies were performed with Sybyl X-1.2 software installed in a Windows 10 environment on a PC with an Intel core i7 CPU, according our previous reports [8-10]. To acquire the best conformers for each molecule, every compound was drawn in ChemDraw and then subjected to a preliminary geometry optimization using MM2 molecular mechanics. Afterwards, the structures were minimized by Tripos force field implemented in Sybyl. Gasteiger-Hückel charges were assigned to each atom. Among the techniques to perform molecular alignment are: 1) atom-by-atom alignments using a common fragment, 2) rigid alignment that minimizes RMS distance, 3) flexible alignments [11] and 4) receptor guided alignments. In the present study, the second option was carried out (Figure S5). The minimum energy conformation of the α,β-unsaturated carbonyl core [obtained from DFT-B3LYP-6-31G(d,p)] was chosen as the common scaffold for alignment. The alignment was carried out using the Distill rigid protocol, as it is implemented in Sybyl.

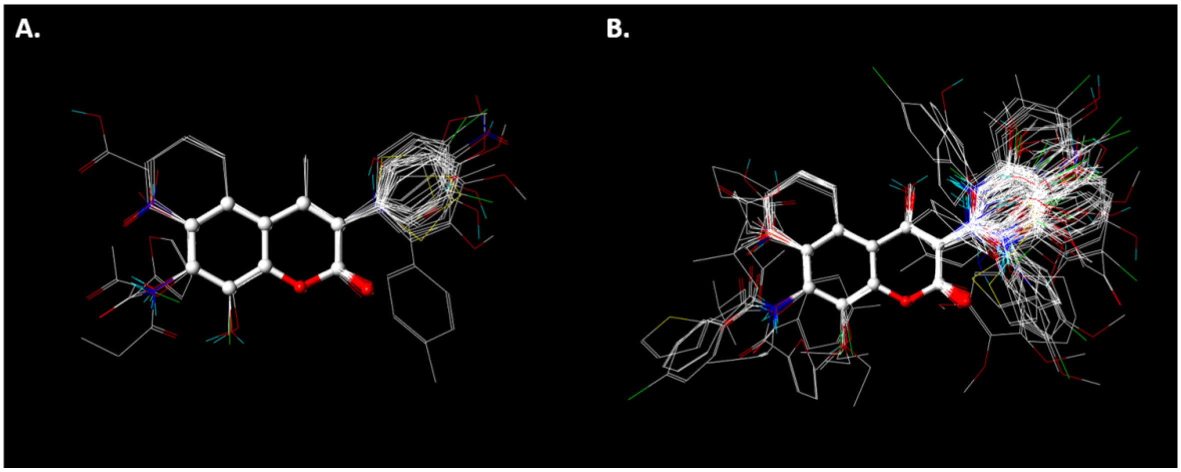

Figure S5. The superimposed structures of all compounds used in the 3D-QSAR model. **A.** Alignment used to MAO-A inhibitors. **B.** Alignment used to MAO-B inhibitors.

CoMSIA field calculation

To derive the CoMFA and CoMSIA descriptor fields, the aligned training set molecules were placed in a three-dimensional cubic lattice with a grid spacing of 2Å in the x, y and z directions such that the entire set was included on it. The CoMFA steric and electrostatic field energies were calculated using a sp3 carbon probe atom with a van der Waals radius of 1.52Å and a charge of +1.0. Cut-off values for both steric and electrostatic fields were set to 30.0 kcal/mol. For CoMSIA analysis, the standard settings (probe with charge +1.0, radius 1Å, hydrophobicity +1.0, H-bond donating +1.0, and H-bond accepting +1.0) were used to calculate five different fields: steric, electrostatic, hydrophobic, donor and acceptor [12]. Gaussian-type distance dependence was used to measure the relative attenuation of the field position of each atom in the lattice and led to a much smoother sampling of the fields around the molecules when compared to CoMFA. The default value of 0.3 was set for attenuation factor α. The results are included in Table S9-S10.

Table S8. Inhibitory activity of coumarin and chromone derivatives against MAO-A and MAO-B.

Compounds	IC_{50} (M)		pIC_{50} (M)	
	MAO-A	MAO-B	MAO-A	MAO-B
**1**	*	$2.84x10^{-7}$	*	6.547
**2**	*	$1.31x10^{-8}$	*	7.884
**3**	*	$8.98x10^{-9}$	*	8.047
**4**	*	$1.61x10^{-7}$	*	6.794
**5**	*	$8.03x10^{-10}$	*	9.096
**6**	*	$1.56x10^{-7}$	*	6.808
**7**	*	$4.30x10^{-6}$	*	5.367
**8**	*	$1.11x10^{-8}$	*	7.957
**9**	*	$3.23x10^{-9}$	*	8.491
**10**	*	$7.12x10^{-9}$	*	8.148
**11**	$3.12x10^{-5}$	$4.89x10^{-6}$	4.506	5.311
**12**	*	$1.71x10^{-8}$	*	7.768
**13**	*	$1.52x10^{-9}$	*	8.818
**14**	$2.49x10^{-5}$	$6.71x10^{-8}$	4.604	7.173
**15**	*	$5.52x10^{-6}$	*	5.258
**16**	*	$1.45x10^{-5}$	*	4.84
**17**	*	$3.10x10^{-10}$	*	9.509
**18**	*	$1.50x10^{-8}$	*	7.824
**19**	*	$2.73x10^{-9}$	*	8.564
**20**	*	$7.40x10^{-10}$	*	9.131
**21**	*	$3.25x10^{-9}$	*	8.488
**22**	*	$5.40x10^{-5}$	*	4.267
**23**	$3.50x10^{-5}$	$6.50x10^{-7}$	4.455	6.187
**24**	$2.16x10^{-5}$	$1.20x10^{-7}$	4.666	6.921
**25**	*	$1.80x10^{-7}$	*	6.745
**26**	*	$6.96x10^{-5}$	*	4.157
**27**	*	$3.20x10^{-5}$	*	4.494
**28**	*	$9.26x10^{-6}$	*	5.033
**29**	*	$4.27x10^{-5}$	*	4.37
**30**	*	$2.79x10^{-6}$	*	5.554
**31**	*	$1.06x10^{-6}$	*	5.975
**32**	*	$2.09x10^{-7}$	*	6.68
**33**	*	$1.49x10^{-7}$	*	6.828
**34**	*	$1.50x10^{-7}$	*	6.824
**35**	*	$3.31x10^{-6}$	*	5.48
**36**	$6.26x10^{-6}$	$7.83x10^{-8}$	5.203	7.106
**37**	$2.16x10^{-5}$	$3.43x10^{-9}$	4.666	8.465

**38**	*	$4.51 x 10^{-9}$	*	8.346
**39**	*	$1.99 x 10^{-8}$	*	7.701
**40**	*	$1.18 x 10^{-5}$	*	4.928
**41**	$4.60 x 10^{-5}$	$1.70 x 10^{-7}$	4.337	6.77
**42**	*	$7.96 x 10^{-6}$	*	5.099
**43**	*	$7.60 x 10^{-7}$	*	6.119
**44**	*	$3.05 x 10^{-5}$	*	4.516
**45**	*	$1.95 x 10^{-6}$	*	5.71
**46**	*	$5.84 x 10^{-5}$	*	4.234
**47**	*	$7.26 x 10^{-5}$	*	4.139
**48**	*	$5.95 x 10^{-6}$	*	5.225
**49**	*	$5.00 x 10^{-5}$	*	4.301
**50**	$3.89 x 10^{-5}$	*	4.41	*
**51**	*	$1.63 x 10^{-6}$	*	5.788
**52**	*	$7.20 x 10^{-8}$	*	7.143
**53**	*	$6.00 x 10^{-9}$	*	8.222
**54**	$1.17 x 10^{-6}$	$3.00 x 10^{-9}$	5.932	8.523
**55**	$3.11 x 10^{-5}$	$1.06 x 10^{-6}$	4.507	5.975
**56**	*	$3.34 x 10^{-5}$	*	4.477
**57**	*	$6.63 x 10^{-6}$	*	5.178
**58**	*	$1.85 x 10^{-7}$	*	6.733
**59**	*	$5.08 x 10^{-6}$	*	5.294
**60**	*	$2.43 x 10^{-5}$	*	4.614
**61**	*	$4.50 x 10^{-8}$	*	7.347
**62**	*	$4.13 x 10^{-7}$	*	6.384
**63**	$2.99 x 10^{-5}$	$3.39 x 10^{-6}$	4.524	5.47
**64**	*	$3.87 x 10^{-10}$	*	9.412
**65**	*	$1.34 \ x 10^{-10}$	*	9.873
**66**	*	$3.20 \ x 10^{-10}$	*	9.495
**67**	*	$6.50 \ x 10^{-10}$	*	9.187
**68**	$1.28 x 10^{-5}$	$3.20 x 10^{-8}$	4.892	7.495
**69**	*	$8.00 x 10^{-9}$	*	8.097
**70**	*	$7.02 x 10^{-6}$	*	5.154
**71**	*	$1.48 x 10^{-6}$	*	5.83
**72**	$4.43 x 10^{-5}$	$1.79 x 10^{-6}$	4.354	5.747
**73**	$1.27 x 10^{-5}$	$7.01 x 10^{-9}$	4.898	8.154
**74**	*	$2.40 x 10^{-9}$	*	8.62
**75**	*	$5.79 x 10^{-8}$	*	7.237
**76**	*	$2.44 x 10^{-9}$	*	8.613
**77**	*	$4.12 x 10^{-8}$	*	7.385
**78**	*	$4.88 x 10^{-8}$	*	7.312
**79**	*	$1.41 x 10^{-6}$	*	5.851
**80**	*	$2.40 x 10^{-6}$	*	5.62

**81**	$2.37x10^{-5}$	$4.60x10^{-7}$	4.626	6.337
**82**	$2.57x10^{-5}$	$5.06x10^{-6}$	4.591	5.296
**83**	*	$3.19x10^{-6}$	*	5.496
**84**	$5.70x10^{-7}$	$5.50x10^{-7}$	6.244	6.26
**85**	*	$3.14x10^{-5}$	*	4.503
**86**	*	$5.52x10^{-6}$	*	5.258
**87**	*	$6.00x10^{-9}$	*	8.222
**88**	$4.18x10^{-6}$	$2.10x10^{-9}$	5.379	8.678
**89**	*	$2.17x10^{-6}$	*	5.664
**90**	*	$2.20x10^{-9}$	*	8.658
**91**	*	$4.10x10^{-9}$	*	8.387
**92**	*	$3.69x10^{-5}$	*	4.433
**93**	*	$1.90x10^{-5}$	*	4.721
**94**	*	$2.27x10^{-6}$	*	5.644
**95**	*	$1.55x10^{-5}$	*	4.81
**96**	*	$2.11x10^{-5}$	*	4.676
**97**	*	$2.25x10^{-5}$	*	4.648
**98**	*	$1.18x10^{-8}$	*	7.928
**99**	*	$7.52x10^{-9}$	*	8.124
**100**	*	$1.39x10^{-8}$	*	7.857
**101**	*	$1.61x10^{-7}$	*	6.794
**102**	*	$1.01x10^{-8}$	*	7.996
**103**	*	$2.97x10^{-7}$	*	6.527
**104**	*	$1.35x10^{-8}$	*	7.87
**105**	*	$4.66x10^{-9}$	*	8.332
**106**	*	$1.14x10^{-8}$	*	7.943
**107**	*	$1.83x10^{-8}$	*	7.738
**108**	*	$4.54x10^{-8}$	*	7.343
**109**	*	$6.22x10^{-7}$	*	6.206
**110**	*	$1.53x10^{-8}$	*	7.815
**111**	*	$7.52x10^{-9}$	*	8.124
**112**	*	$5.07x10^{-9}$	*	8.295
**113**	*	$4.54x10^{-8}$	*	7.343
**114**	*	$1.11x10^{-8}$	*	7.955
**115**	*	$6.22x10^{-7}$	*	6.206
**116**	*	$5.95x10^{-9}$	*	8.225
**117**	*	$4.72x10^{-8}$	*	7.326
**118**	*	$9.03x10^{-9}$	*	8.044
**119**	*	$2.29x10^{-7}$	*	6.641
**120**	*	$1.94x10^{-8}$	*	7.712
**121**	*	$1.89x10^{-8}$	*	7.724
**122**	*	$2.14x10^{-8}$	*	7.671
**123**	*	$1.71x10^{-8}$	*	7.767

**124**	*	4.20×10^{-9}	*	8.377
**125**	*	7.82×10^{-8}	*	7.107
**126**	*	1.52×10^{-7}	*	6.819
**127**	*	4.54×10^{-8}	*	7.343
**128**	*	5.13×10^{-7}	*	6.29
**129**	*	4.18×10^{-8}	*	7.379
**130**	*	2.18×10^{-8}	*	7.662
**131**	*	3.94×10^{-9}	*	8.405
**132**	*	1.14×10^{-7}	*	6.945
**133**	*	2.11×10^{-7}	*	6.676
**134**	*	1.03×10^{-8}	*	7.987
**135**	*	6.74×10^{-7}	*	6.171
**136**	*	4.70×10^{-6}	*	5.328
**137**	*	6.50×10^{-9}	*	8.187
**138**	*	1.07×10^{-5}	*	4.971
**139**	*	2.50×10^{-6}	*	5.602
**140**	*	1.32×10^{-7}	*	6.879
**141**	*	8.10×10^{-8}	*	7.092
**142**	1.76×10^{-5}	*	4.754	*
**143**	7.62×10^{-6}	8.50×10^{-6}	5.118	5.071
**144**	1.32×10^{-6}	4.75×10^{-6}	5.879	5.323
**145**	6.21×10^{-6}	*	5.207	*
**146**	9.16×10^{-6}	1.40×10^{-7}	5.038	6.854
**147**	6.08×10^{-6}	2.18×10^{-5}	5.216	4.663
**148**	*	9.50×10^{-7}	*	6.022
**149**	*	1.20×10^{-5}	*	4.922
**150**	*	1.09×10^{-6}	*	5.963
**151**	*	3.97×10^{-6}	*	5.401
**152**	*	8.05×10^{-6}	*	5.094
**153**	2.57×10^{-5}	*	4.59	*
**154**	9.67×10^{-5}	*	4.015	*
**155**	2.45×10^{-5}	1.16×10^{-5}	4.611	4.937
**156**	4.59×10^{-5}	4.40×10^{-7}	4.339	6.357
**157**	*	2.50×10^{-7}	*	6.602
**158**	7.82×10^{-5}	In	4.107	In
**159**	*	3.60×10^{-7}	*	6.444
**160**	*	9.00×10^{-7}	*	6.046
**161**	*	3.10×10^{-7}	*	6.509
**162**	*	1.59×10^{-6}	*	5.799
**163**	3.01×10^{-5}	5.01×10^{-6}	4.521	5.3

* Inactive compound; $IC_{50} > 100$ μM.

Table S9. Field combination of CoMFA and CoMSIA models of MAO-A inhibitors.

	F. C.	q^2	N	SEP	SEE	r_{ncv}^2	F	Field Contribution				
								S	E	H	D	A
CoMFA	S	0.583	7	0.462	0.03	0.998	1148.4	1				
	E	0.42	4	0.495	0.167	0.934	60.1		1			
	**SE**	**0.612**	**3**	**0.393**	**0.161**	**0.935**	**85.9**	**0.656**	**0.344**			
CoMSIA	S	0.375	3	0.499	0.272	0.814	26.3	1				
	E	0.056	2	0.597	0.404	0.567	12.5		1			
	H	0.169	2	0.56	0.319	0.73	25.6			1		
	D	-0.401	1	0.709	0.417	0.516	21.3				1	
	A	0.253	4	0.727	0.331	0.74	12.1					1
	SE	0.395	7	0.557	0.073	0.989	188.4	0.489	0.511			
	**SEH**	**0.523**	**5**	**0.462**	**0.098**	**0.979**	**146.2**	**0.259**	**0.358**	**0.383**		
	SEHD	0.359	13	0.758	0.003	1	57426	0.185	0.264	0.338	0.213	
	SEHA	0.493	14	0.721	0.002	1	183980	0.203	0.298	0.351		0.148
	SED	0.101	3	0.599	0.24	0.856	35.6	0.245	0.327		0.428	
	SEA	0.26	4	0.559	0.161	0.938	64.8	0.376	0.404			0.22
	SEDA	0.035	2	0.603	0.28	0.792	36.2	0.211	0.249		0.398	0.143
	SH	0.236	2	0.537	0.309	0.747	28.1	0.374		0.626		
	SD	-0.041	5	0.683	0.167	0.938	48.2	0.483			0.517	
	SA	0.225	5	0.589	0.144	0.954	66.1	0.557				0.443
	SHD	0.233	7	0.627	0.054	0.994	351.6	0.218		0.409	0.373	
	SHA	0.279	9	0.656	0.018	0.999	2363.6	0.244	0.44			0.315
	SDA	-0.112	5	0.706	0.167	0.938	48.1	0.379			0.406	0.215
	SHDA	0.137	8	0.69	0.037	0.997	637.6	0.196		0.391	0.286	0.128
	EH	0.532	15	0.748	0.001	1	217372		0.473	0.527		
	ED	-0.132	2	0.654	0.347	0.681	20.3		0.378		0.622	
	EA	-0.046	1	0.612	0.506	0.286	8		0.675			0.325
	EHD	0.231	14	0.888	0.002	1	99621.2		0.309	0.43	0.261	
	EHA	0.4	15	0.847	0.001	1	974200		0.348	0.471		0.182
	EDA	-0.141	2	0.656	0.338	0.697	21.8		0.303		0.501	0.197
	EHDA	0.175	13	0.86	0.004	1	30157.6		0.279	0.404	0.214	0.103
	HD	0.054	6	0.672	0.075	0.988	209			0.563	0.437	
	HA	0.173	9	0.703	0.022	0.999	1646.2			0.622		0.378
	HDA	-0.003	6	0.692	0.092	0.982	137.9			0.477	0.341	0.182
	DA	-0.345	2	0.713	0.348	0.679	20.1				0.693	0.307
	ALL	0.296	13	0.794	0.003	1	46419	0.169	0.244	0.322	0.182	0.083

F.C., is the field combination; q^2, the square of the LOO cross-validation (CV) coefficient; N, the optimum number of components; SEP, standard error of prediction; SEE, standard error of estimation of non CV analysis; r_{ncv}^2, square of the non CV coefficient; F, F-test value;

S, E, H, D and A are the steric, electrostatic, hydrophobic, hydrogen-bond donor, and hydrogen-bond acceptor contributions respectively.

Table S10. Field combination of CoMFA and CoMSIA models of MAO-B inhibitors.

	F.C.	q^2	N	SEP	SEE	r_{ncv}^2	F	Field Contribution				
								S	E	H	D	A
CoMFA	S	0.768	5	0.686	0.372	0.932	281.4	1				
	E	0.428	4	1.073	0.86	0.632	44.7		1			
	**SE**	**0.841**	**3**	**0.562**	**0.474**	**0.887**	**275.9**	**0.676**	**0.324**			
CoMSIA	S	0.729	5	0.742	0.543	0.855	121.3	1				
	E	0.345	3	1.142	0.961	0.536	40.4		1			
	H	0.613	3	0.879	0.729	0.734	96.3			1		
	D	0.296	5	1.196	1.035	0.472	18.4				1	
	A	0.214	4	1.258	1.125	0.371	15.3					1
	SE	0.696	3	0.779	0.605	0.816	155.6	0.547	0.453			
	SEH	0.761	4	0.694	0.469	0.891	212.2	0.281	0.319	0.4		
	**SEHD**	**0.772**	**4**	**0.677**	**0.469**	**0.891**	**212.2**	**0.23**	**0.215**	**0.334**	**0.221**	
	SEHA	0.769	4	0.682	0.489	0.881	192.9	0.254	0.267	0.371		0.107
	SED	0.715	5	0.76	0.508	0.873	141.3	0.381	0.296		0.323	
	SEA	0.691	5	0.793	0.506	0.874	142.9	0.441	0.358			0.201
	SEDA	0.71	6	0.771	0.464	0.895	145	0.343	0.258		0.269	0.13
	SH	0.724	3	0.741	0.588	0.826	166.7	0.409		0.591		
	SD	0.749	7	0.721	0.472	0.892	119.6	0.49			0.51	
	SA	0.745	8	0.73	0.406	0.921	145.8	0.598				0.402
	SHD	0.758	4	0.698	0.511	0.87	174.5	0.275		0.407	0.319	
	SHA	0.74	8	0.738	0.32	0.951	242.7	0.325		0.455		0.221
	SDA	0.764	7	0.7	0.446	0.904	136.5	0.418			0.385	0.197
	SHDA	0.764	5	0.692	0.457	0.897	179.5	0.249		0.355	0.291	0.105
	EH	0.678	3	0.801	0.64	0.794	135		0.351	0.649		
	ED	0.417	4	1.083	0.854	0.638	45.8		0.518		0.638	
	EA	0.36	2	1.124	1.026	0.466	46.3		0.611			0.389
	EHD	0.708	4	0.767	0.577	0.835	131.2		0.25	0.476	0.274	
	EHA	0.682	2	0.871	0.688	0.76	168.1		0.307	0.547		0.146
	EDA	0.412	4	1.088	0.856	0.636	45.3		0.442		0.379	0.179
	EHDA	0.702	5	0.778	0.54	0.857	123.1		0.223	0.433	0.256	0.088
	HD	0.686	4	0.795	0.621	0.808	109.7			0.597	0.403	
	HA	0.638	3	0.85	0.694	0.758	109.8			0.771		0.689
	HDA	0.694	5	0.788	0.572	0.839	107.4			0.525	0.347	0.128
	DA	0.301	5	1.191	0.984	0.523	22.6				0.684	0.316
	ALL	0.767	4	0.684	0.489	0.881	192.6	0.221	0.194	0.326	0.193	0.232

F.C., is the field combination; q^2, the square of the LOO cross-validation (CV) coefficient; N, the optimum number of components; SEP, standard error of prediction; SEE, standard error of estimation of non-CV analysis; r_{ncv}^2, square of the non CV coefficient; F, F-test value;

S, E, H, D and A are the steric, electrostatic, hydrophobic, hydrogen-bond donor, and hydrogen-bond acceptor contributions respectively.

Table S11. Summary of the best CoMFA and CoMSIA models of MAO-A and MAO-B inhibitors.

**MAO**	**Model**	**q^2**	**N**	**SEP**	**SEE**	**r_{ncv}^2**	**F**	**Field Contribution**					**PRESS**	**SD**	**r^2_{Pred}**
								**S**	**E**	**H**	**D**	**A**			
A	CoMFA-SE	0.612	3	0.393	0.161	0.935	85.9	0.656	0.344				0.1274	0.5194	0.755
	CoMSIA-SEH	0.523	5	0.462	0.098	0.979	146.2	0.259	0.358	0.383			0.1021	0.6898	0.852
B	CoMFA-SE	0.841	3	0.562	0.474	0.887	275.9	0.676	0.324				13.293	66.556	0.8
	CoMSIA-SEHD	0.772	4	0.677	0.469	0.891	212.2	0.23	0.215	0.334	0.221		8.5762	68.058	0.874

F.C., is the field combination; q^2, the square of the LOO cross-validation (CV) coefficient; N, the optimum number of components; SEP, standard error of prediction; SEE, standard error of estimation of non-CV analysis; r_{ncv}^2, square of the non-CV coefficient; F, F-test value; S, E, H, D and A are the steric, electrostatic, hydrophobic, hydrogen-bond donor, and hydrogen-bond acceptor contributions respectively.

Table S12. Comparison between experimental and predicted values of the CoMFA and CoMSIA models obtained from MAO-A inhibitors.

Compound	Experimental pIC_{50}	Calculated pIC_{50}			
		CoMFA-SE	Residual	CoMSIA-SEH	Residual
**11**	4.506	4.417	0.089	4.417	0.089
**14**	4.604	4.521	0.083	4.521	0.083
**23**	4.455	4.373	0.082	4.373	0.082
**24**	4.666	4.569	0.097	4.569	0.097
**36**	5.203	5.378	-0.175	5.378	-0.175
**37**[a,b]	4.666	4.786	-0.121	4.627	0.039
**41**	4.338	4.241	0.097	4.241	0.097
**50**[a,b]	4.41	4.413	-0.004	4.427	-0.018
**54**	5.932	5.829	0.103	5.829	0.103
**55**	4.507	4.71	-0.203	4.71	-0.203
**63**	4.524	4.464	0.06	4.464	0.06
**68**[a,b]	4.892	4.822	0.07	4.895	-0.003
**82**[a,b]	4.354	4.639	-0.285	4.496	-0.142
**73**	4.898	4.99	-0.092	4.99	-0.092
**81**[a,b]	4.626	4.735	-0.109	4.824	-0.198
**82**	4.591	4.538	0.053	4.538	0.053
**84**	6.244	6.066	0.178	6.066	0.178
**88**	5.379	5.647	-0.268	5.647	-0.268
**142**	4.754	4.975	-0.221	4.975	-0.221
**143**	5.118	5.143	-0.025	5.143	-0.025
**144**	5.879	5.559	0.32	5.559	0.32
**145**[a,b]	5.207	5.156	0.051	5.217	-0.01
**146**	5.038	4.949	0.089	4.949	0.089
**147**	5.216	5.387	-0.171	5.387	-0.171
**153**	4.59	4.459	0.131	4.459	0.131
**154**	4.015	4.128	-0.113	4.128	-0.113
**155**[a,b]	4.611	4.511	0.1	4.426	0.185
**156**[a,b]	4.339	4.293	0.046	4.258	0.08
**158**	4.107	4.192	-0.085	4.192	-0.085
**163**	4.521	4.551	-0.03	4.551	-0.03

[a] = Test set of CoMFA model. [b] = Test set of CoMSIA model.

Table S13. Comparison between experimental and predicted values of the CoMFA and CoMSIA models obtained from MAO-B inhibitors.

Compound	Experimental pIC_{50}	Calculated pIC_{50}			
		CoMFA-SE	Residual	CoMSIA-SEHD	Residual
**1**	6.547	6.333	0.214	6.333	0.214
**2**	7.884	8.038	-0.154	8.038	-0.154
**3**	8.047	8.558	-0.511	8.558	-0.511
**4**[a,b]	6.794	6.82	-0.025	6.737	0.057
**5**[a,b]	9.096	8.388	0.708	8.195	0.9
**6**[a,b]	6.808	6.928	-0.12	7.091	-0.283
**7**[a,b]	5.367	5.359	0.007	5.463	-0.096
**8**[a,b]	7.957	7.736	0.221	7.85	0.106
**9**[a,b]	8.491	8.458	0.033	8.532	-0.041
**10**	8.148	8.59	-0.442	8.59	-0.442
**11**	5.311	5.5	-0.189	5.5	-0.189
**12**	7.768	8.064	-0.296	8.064	-0.296
**13**[a,b]	8.818	8.783	0.035	8.483	0.335
**14**[a,b]	7.173	7.253	-0.079	7.109	0.064
**15**	5.258	5.717	-0.459	5.717	-0.459
**16**	4.84	5.516	-0.676	5.516	-0.676
**17**	9.509	8.692	0.817	8.692	0.817
**18**	7.824	8.419	-0.595	8.419	-0.595
**19**	8.564	8.691	-0.127	8.691	-0.127
**20**[a,b]	9.131	8.15	0.981	8.384	0.747
**21**[a,b]	8.488	8.54	-0.052	8.365	0.123
**22**	4.267	5.146	-0.879	5.146	-0.879
**23**	6.187	5.871	0.316	5.871	0.316
**24**	6.921	7.306	-0.385	7.306	-0.385
**25**	6.745	7.268	-0.523	7.268	-0.523
**26**	4.157	6.127	-1.97	6.127	-1.97
**27**[a,b]	4.494	5.949	-1.455	5.281	-0.787
**28**[a,b]	5.033	6.111	-1.077	5.802	-0.769
**29**	4.37	6.001	OUT	5.288	OUT
**30**	5.554	5.479	0.075	5.479	0.075
**31**	5.975	5.374	0.601	5.374	0.601
**32**	6.68	6.513	0.167	6.513	0.167
**33**[a,b]	6.828	6.881	-0.053	6.733	0.095
**34**	6.824	6.633	0.191	6.633	0.191
**35**[a,b]	5.48	5.391	0.089	5.249	0.231
**36**	7.106	7.681	-0.575	7.681	-0.575
**37**	8.465	8.312	0.153	8.312	0.153

**38**	8.346	8.424	-0.078	8.424	-0.078
**39**	7.701	8.04	-0.339	8.04	-0.339
**40**	4.928	5.309	-0.381	5.309	-0.381
**41**	6.77	6.918	-0.148	6.918	-0.148
**42**	5.099	4.695	0.404	4.695	0.404
**43**	6.119	5.974	0.145	5.974	0.145
**44**[a,b]	4.516	4.531	-0.015	4.617	-0.101
**45**	5.71	5.997	-0.287	5.997	-0.287
**46**	4.234	4.426	-0.192	4.426	-0.192
**47**	4.139	4.294	-0.155	4.294	-0.155
**48**	5.225	4.944	0.281	4.944	0.281
**49**	4.301	4.584	-0.283	4.584	-0.283
**51**	5.788	5.399	0.389	5.399	0.389
**52**[a,b]	7.143	7.135	0.008	7.259	-0.116
**53**[a,b]	8.222	8.231	-0.009	7.88	0.341
**54**[a,b]	8.523	7.928	0.595	7.713	0.81
**55**	5.975	5.521	0.454	5.521	0.454
**56**	4.477	4.779	-0.302	4.779	-0.302
**57**[a,b]	5.178	5.889	-0.71	6.316	-1.137
**58**[a,b]	6.733	6.528	0.205	6.634	0.099
**59**[a,b]	5.294	5.378	-0.084	5.46	-0.166
**60**[a,b]	4.614	4.742	-0.127	4.25	0.365
**61**	7.347	7.053	0.294	7.053	0.294
**62**	6.384	6.542	-0.158	6.542	-0.158
**63**	5.47	5.551	-0.081	5.551	-0.081
**64**	9.412	8.569	0.843	8.569	0.843
**65**	9.873	8.332	1.541	8.332	1.541
**66**	9.495	8.901	0.594	8.901	0.594
**67**	9.187	7.72	1.467	7.72	1.467
**68**	7.495	7.287	0.208	7.287	0.208
**69**	8.097	8.459	-0.362	8.459	-0.362
**70**[a,b]	5.154	5.841	-0.687	5.487	-0.333
**71**[a,b]	5.83	6.779	-0.95	6.073	-0.243
**72**	5.747	5.747	0	5.747	0
**73**	8.154	8.619	-0.465	8.619	-0.465
**74**	8.62	8.184	0.436	8.184	0.436
**75**[a,b]	7.237	7.23	0.007	7.215	0.022
**76**[a,b]	8.613	7.777	0.836	7.574	1.039
**77**	7.385	8.064	-0.679	8.064	-0.679
**78**	7.312	7.624	-0.312	7.624	-0.312
**79**	5.851	5.213	0.638	5.213	0.638
**80**	5.62	5.429	0.191	5.429	0.191
**81**	6.337	6.473	-0.136	6.473	-0.136

**82**	5.296	5.342	-0.046	5.342	-0.046
**83**[a,b]	5.496	5.75	-0.254	5.881	-0.385
**84**	6.26	6.22	0.04	6.22	0.04
**85**	4.503	4.992	-0.489	4.992	-0.489
**86**	5.258	5.617	-0.359	5.617	-0.359
**87**[a,b]	8.222	8.193	0.029	7.491	0.731
**88**	8.678	8.6	0.078	8.6	0.078
**89**	5.664	6.092	-0.428	6.092	-0.428
**90**	8.658	8.033	0.625	8.033	0.625
**91**	8.387	8.047	0.34	8.047	0.34
**92**	4.433	6.107	OUT	5.775	OUT
**93**[a,b]	4.721	4.721	0	4.956	-0.235
**94**	5.644	5.357	0.287	5.357	0.287
**95**	4.81	4.67	0.14	4.67	0.14
**96**	4.676	4.815	-0.139	4.815	-0.139
**97**[a,b]	4.648	5.77	-1.122	5.12	-0.472
**98**[a,b]	7.928	8.026	-0.098	7.912	0.016
**99**[a,b]	8.124	8.166	-0.042	8.039	0.085
**100**	7.857	7.479	0.378	7.479	0.378
**101**	6.794	6.182	0.612	6.182	0.612
**102**	7.996	7.976	0.02	7.976	0.02
**103**[a,b]	6.527	6.353	0.174	6.242	0.285
**104**	7.87	8.198	-0.328	8.198	-0.328
**105**[a,b]	8.332	7.456	0.876	8.051	0.281
**106**	7.943	7.448	0.495	7.448	0.495
**107**	7.738	7.711	0.027	7.711	0.027
**108**	7.343	7.581	-0.238	7.581	-0.238
**109**[a,b]	6.206	6.501	-0.294	6.093	0.114
**110**	7.815	8.081	-0.266	8.081	-0.266
**111**	8.124	8.755	-0.631	8.755	-0.631
**112**	8.295	8.111	0.184	8.111	0.184
**113**	7.343	7.711	-0.368	7.711	-0.368
**114**[a,b]	7.955	7.848	0.108	8.132	-0.176
**115**	6.206	6.176	0.03	6.176	0.03
**116**	8.225	8.359	-0.134	8.359	-0.134
**117**	7.326	7.973	-0.647	7.973	-0.647
**118**	8.044	8.133	-0.089	8.133	-0.089
**119**[a,b]	6.641	6.384	0.257	6.7	-0.059
**120**[a,b]	7.712	7.851	-0.139	7.383	0.328
**121**	7.724	7.892	-0.168	7.892	-0.168
**122**	7.671	7.889	-0.218	7.889	-0.218
**123**	7.767	7.991	-0.224	7.991	-0.224
**124**[a,b]	8.377	8.42	-0.043	8.472	-0.095

**125**	7.107	6.983	0.124	6.983	0.124
**126**	6.819	6.229	0.59	6.229	0.59
**127**	7.343	6.892	0.451	6.892	0.451
**128**	6.29	6.062	0.228	6.062	0.228
**129[a,b]**	7.379	7.361	0.018	7.407	-0.028
**130**	7.662	7.47	0.192	7.47	0.192
**131**	8.405	8.183	0.222	8.183	0.222
**132[a,b]**	6.945	6.843	0.102	6.907	0.038
**133**	6.676	6.2	0.476	6.2	0.476
**134**	7.987	8.359	-0.372	8.359	-0.372
**135**	6.171	6.076	0.095	6.076	0.095
**136**	5.328	5.44	-0.112	5.44	-0.112
**137**	8.187	7.554	0.633	7.554	0.633
**138**	4.971	4.571	0.4	4.571	0.4
**139**	5.602	5.11	0.492	5.11	0.492
**140**	6.879	6.378	0.501	6.378	0.501
**141**	7.092	7.749	-0.657	7.749	-0.657
**142**	5.071	4.978	0.093	4.978	0.093
**144[a,b]**	5.323	4.716	0.607	4.922	0.402
**146**	6.854	5.109	OUT	6.276	OUT
**147**	4.663	4.907	-0.244	4.907	-0.244
**148**	6.022	5.747	0.275	5.747	0.275
**149**	4.922	5.077	-0.155	5.077	-0.155
**150[a,b]**	5.963	5.935	0.028	6.005	-0.042
**151**	5.401	5.957	-0.556	5.957	-0.556
**152[a,b]**	5.094	6.427	-1.333	5.415	-0.32
**155[a,b]**	4.937	5.936	-0.998	5.731	-0.793
**156**	6.357	6.098	0.259	6.098	0.259
**157**	6.602	6.279	0.323	6.279	0.323
**159**	6.444	6.318	0.126	6.318	0.126
**160**	6.046	5.981	0.065	5.981	0.065
**161**	6.509	6.314	0.195	6.314	0.195
**162**	5.799	6.069	-0.27	6.069	-0.27
**163**	5.3	5.436	-0.136	5.436	-0.136

[a] = Test set of CoMFA model. [b] = Test set of CoMSIA model.

Internal validation and Partial Least Squares (PLS) analysis

PLS analysis was used to construct a linear correlation between the CoMFA and CoMSIA descriptors (independent variables) and the activity values (dependent variables) [30]. To select the best model, the cross-validation analysis was performed by using the LOO method (and SAMPLS), which generates the square of the cross-validation coefficient (q^2) and the optimum number of components (N). The non-cross-validation was performed with a column filter value of 2.0 in order to speed up the analysis and reduce the noise. The q^2, which is a measure of the internal quality of the models was obtained according to the following equation (1):

$$q^2 = 1 - \frac{\sum(y_i - y_{pred})^2}{\sum(y_i - \bar{y})^2} \quad (1)$$

Where y_i, $\bar{y}$, and y_{pred} are observed, mean, and predicted activity in the training set, respectively.

External validation

The models were subjected to external validation criteria according to the proposed test by Golbraikh and Tropsha [31,32], which considers a QSAR model predictive, if the following conditions are satisfied:

$$q^2 > 0.5 \quad (2)$$

$$r^2 > 0.6 \quad (3)$$

$$0.85 \leq k \leq 1.15 \text{ or } 0.85 \leq k' \leq 1.15 \quad (4)$$

It has been demonstrated [33] that all of the above criteria are indeed necessary to adequately assess the predictive ability of a QSAR model.

Furthermore, the external predictive power of the developed 3D-QSAR models using the test set was examined by considering r_m^2 metrics as shown below [34]:

$$r_m^2 = r^2\left(1 - \left|\sqrt{r^2 - r_0^2}\right|\right) \quad (5)$$

Where r^2 and r_0^2 are squared correlation coefficients between the observed and predicted activities of the test set with and without intercept, respectively. For a significant external model validation, the value of q^2 should be higher than 0.5.

Additionally, the following descriptors were calculated:

$$Q_{F1}^2 = 1 - \frac{\sum_{i=1}^{n_{EXT}}(y_i - \hat{y}_i)^2}{\sum_{i=1}^{n_{EXT}}(y_i - \bar{y}_{TR})^2} \quad (6)$$

$$Q_{F2}^2 = 1 - \frac{\sum_{i=1}^{n_{EXT}}(y_i - \hat{y}_i)^2}{\sum_{i=1}^{n_{EXT}}(y_i - \bar{y}_{EXT})^2} \quad (7)$$

$$CCC = \frac{2\sum_{i=1}^{n_{EXT}}(y_i - \bar{y})(\hat{y}_i - \bar{\hat{y}})}{\sum_{i=1}^{n_{EXT}}(y_i - \bar{y})^2 + \sum_{i=1}^{n_{EXT}}(\hat{y}_i - \bar{\hat{y}})^2 + n_{EXT}\left(\bar{y} - \bar{\hat{y}}\right)^2} \quad (8)$$

$$\Delta r_m^2 = |r_m^2 - r_m'^2| \quad (9)$$

$$MAE = \frac{\sum_{i=1}^{n_{EXT}}|y_i - \hat{y}_i|}{n_{EXT}} \quad (10)$$

Where, TR = training set, EXT = external prediction set, y_i= experimental data values, $\hat{y}_i$ = predicted data values, $\bar{y}$ = average of the experimental data values, $\bar{\hat{y}}$= average of the predicted data values. Finally, r_m^2 is calculated using the experimental values on the ordinate axis, while $r'_m{}^2$ using them on the abscissa.

Table S14. Summary of external validation parameters for CoMSIA.

Condition	Parameters	Threshold value	CoMFA MAO-A	CoMFA MAO-B	CoMSIA MAO-A	CoMSIA MAO-B
**1**	q^2	>0.5	0.612	0.841	0.523	0.772
**2**	r^2	>0.6	0.805	0.808	0.853	0.911
**3a**	r_0^2	Close to value of r^2	0.999	0.990	0.999	0.995
**3b**	$r'_0{}^2$	Close to value of r^2	0.999	0.990	0.999	0.995
**4a**	k	$0.85 < k < 1.15$	1.006	1.003	1.002	0.992

**4b**	k^2	0.85 < k'< 1.15	0.993	0.988	0.998	1.003
**5**	$\|r_0^2 - r'_0{}^2\|$	<0.3	0.000	0.00	0.000	0.00
**6**	r_m^2	>0.5	0.450	0.463	0.527	0.647
**7**	Δr_m^2	< 0.2	0.000	0.183	0.000	0.000
**8**	Q_{F1}^2	> 0.7	0.877	0.792	0.901	0.890
**9**	Q_{F2}^2	> 0.7	0.792	0.791	0.833	0.890
**10**	*MAE*	<0.3	0.098	0.349	0.084	0.3569
**11**	CCC	> 0.85	0.890	0.873	0.928	0.933

q^2 = the square of the LOO cross-validation (CV) coefficient; r^2 is the regression coefficient for the test set exclusively; r_0^2 and k are the correlation coefficient between the actual and predicted activities for test set and the respective slope of regression; and $r'_0{}^2$ and *k'* are the correlation coefficient between the predicted and actual activities for test set and the respective slope of regression. r_m^2 is the r^2 metrics calculated using (x,y), the $r'_m{}^2$ is calculated using (y, x), and the Δr_m^2 is the difference between r_m^2 and $r'_m{}^2$. The other parameters are defined below.

Applicability domain calculation

The AD was evaluated based on the simple standardization method reported by Roy et al. [31]. First, each descriptor "i" for each compound "k" is standardized (S_{ik}). Every compound must have a maximum value $[S_i]_{max(k)} \leq 3$. In the case that $[S_i]_{max(k)} > 3$ and its minimum value $[s_i]_{min(k)} < 3$, then the $S_{new(k)}$ parameter must be calculated and has to fulfill the condition: $S_{new(k)} = \bar{S}_k + 1.28 * \sigma_{S_k}$, where $\bar{S}_k$ is the mean of *Sik* values for compound *k* and σ_{S_k} is the standard deviation for such values. The software is available free of charge on the authors' website: http://dtclab.webs.com/software-tools and http://teqip.jdvu.ac.in/QSAR_Tools/.

Molecular Docking

The crystalline structures of the human MAOs (A and B isoforms) used for the docking study were downloaded from Protein Data Bank: PDB code 2Z5Y [35] and PDB code 1GOS [36]. The 3D structures of the compounds 3-(3',4'-dihydroxyphenyl)benzo[*f*]coumarin (**84**), 3-(3'-bromophenyl)-6-methylcoumarin (**65**), 3-phenylcoumarin, *trans*-6-styrylcoumarins, *trans*-resveratrol and *trans*-stilbene were generated with the ChemDraw program. The molecular docking was carried out according previous report of our research group [37]. Using the AutoDockTools software, the rotatable bonds of each ligand were assigned, the polar hydrogen atoms were added, and the water molecules were removed from the PDB file of the protein. The docking of each ligand was done using AutoDock vina script [8], with a grid box of 10x10x10 Å(1000 Å), and with a space of 0.375 Å centered in the active site (coordinates MAO-A x= -40.872, y= -26.701, z= -13.711, and coordinates MAO-B x= 57.026, y= 150.786, z= 22.151). The molecular docking results were processed using Pymol [38] and LigPlot+ [39,40] to identify the interactions between the ligands and MAO-A or MAO-B, as well as to generate the 3D representation of the studied compounds.

Molecular dynamics simulations

Molecular dynamics simulations were performed with Gromacs 2020.4 version to evaluate the stability of the protein-enzyme ligand complex formed by 3-phenylcoumarin, 3-(3'-bromophenyl)-6-methylcoumarin, *trans*-6-styrylcoumarin, and *trans*-resveratrol with MAO-B [41]. Input files required for MD simulation were created via the CHARMM-GUI server [42]. Protein and ligand topology files were created using the Charmm36m force field. A rectangular water box was created at a distance of 10 Å from the protein with the TIP3 water model [43]. The system was neutralized by adding 0.15 M KCl and the energy of the system was minimized. It was equilibrated with 0.5 ns duration NVT/NPT steps and 100 ns MD simulation of 1000 frame was performed to 2 fs. RMSD and RMSF measurements were performed with gmx script. Binding free energy MMPBSA calculations were made between 80 ns and 100 ns with the g_mmpbsa script [44].

ADDITIONAL RESULTS

3D-QSAR study

Figure S6 shows the graphs of experimental activity *versus* predictive activity for both CoMFA and CoMSIA models. The gray line corresponds to the line $y = x$, while the red line is the regression line for the test set of each model. CoMFA models tend to underestimate predictions at high activity values. The best fit for linearity has been obtained for the CoMSIA models for both MAO-A and MAO-B.

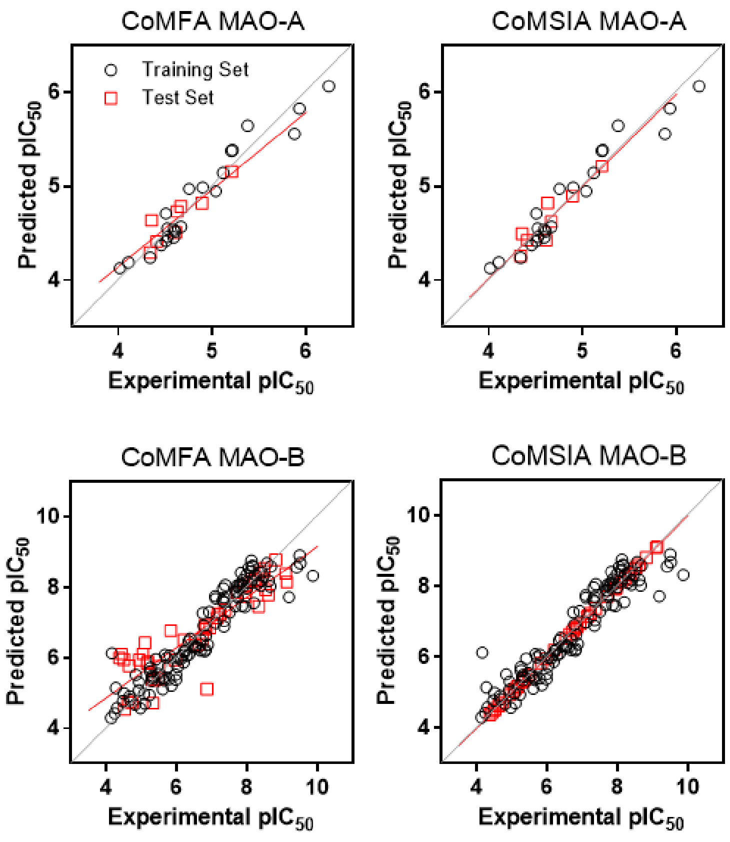

Figure S6. Plots of experimental *versus* predicted pIC_{50} in MAO-A and MAO-B for CoMFA and CoMSIA models.

To subject the models to a rigorous analysis of their adjustment to predictive linearity, r^2_0 values and other descriptors such as q^2_{F1}, q^2_{F2}, CCC, etc., were calculated. The values are reported in Table S14. The applicability domain was calculated according to Roy's standardization method. It

was found that compounds **93** and **146** were outside the domain of applicability of the MAO-B CoMSIA model. These compounds do not present large structural differences with the other molecules in the study. Therefore, the observed deviation could be due to conformational reasons. The summary of the best models is shown in Table S11. There is a high similarity between the steric and electrostatic contributions of the MAO-A and MAO-B CoMFA models (~65% and ~35% of contribution, respectively). However, the MAO-B CoMSIA model shows an important contribution from hydrogen-bond donor groups (22.1%). This is not observed for the MAO-A model. This leads us to conclude that there are different steric and electronic requirements to achieve affinity for both MAO-A and MAO-B.

Steric map of MAO-A inhibitors

The steric contour maps of the CoMFA and CoMSIA analysis shows great similarities, mainly in the region of the 2-aryl ring linked to the benzo[*f*]coumarin scaffold (Figure S7A-C). In this ring, bulky substituents (e.g., hydroxyl or 2-thiophenyl groups, or bromine atoms) that are close to the green polyhedron at *meta* and *para* positions may favor the MAO-A inhibitory activity. This is reflected in the activities of compounds **143** to **147** ($pIC_{50} > 5.0$). However, when some of these substituents are replaced by even bulkier groups (e.g., methoxy groups, etc.), the substituents project into the yellow polyhedron, causing a decrease in the MAO-A inhibitory activity. This occurs in the case of compounds **11**, **37**, **55**, **72** and **73** ($pIC_{50} < 5.0$). Additionally, near to position 6 of the 3-arylbenzo[*f*]coumarin scaffold, a yellow polyhedron is projected (Figure S7C), which implies that a bulky substituent near this area may cause a decrease in the MAO-A inhibitory activity ($pIC_{50} < 5.0$). This is observed for compounds **72**, **73**, **153-156**, **158** and **163**, which have alkylamine and arylamine fragments that project into this yellow polyhedron.

Electrostatic map of MAO-A inhibitors

The electrostatic contour maps obtained from the CoMFA and CoMSIA analysis shows similarities, mainly in the aryl ring linked to position 2 of the benzo[*f*]coumarin scaffold (Figure S7B-D). In this ring, a blue polyhedron is projected in the *meta* and *para* carbons, indicating that the loss of electron density in these carbons may favor the MAO-A inhibitory activity. Additionally, a red polyhedron is projected in the outer part of the blue polyhedron, indicating that electron-rich substituents favor the MAO-A inhibitory activity. Then, considering these two

characteristics, the electronegative substituents attached to the *meta* and *para* positions cause a lost in the electron density, favoring the MAO-A inhibitory activity. This is observed for compounds **54**, **84**, **88**, **145** and **146** (pIC_{50} > 5.0). On the other hand, the CoMFA analysis shows that close to positions 6, 7, and 8 of the benzo[*f*]coumarin scaffold (Figure S7B), a red polyhedron is projected, indicating that the presence of an electron-rich substituent may favor the MAO-A inhibitory activity. The CoMSIA analysis (Figure S7D) shows a red polyhedron close to position 8 of the benzo[*f*]coumarin scaffold, indicating that an electron-rich substituent favors the MAO-A inhibitory activity, as shown for compounds **143** to **147**.

Hydrophobic map of MAO-A inhibitors

The hydrophobic contour map analysis shows that only the hydrophobic or hydrophilic character of the substituents attached to the 3-aryl ring causes changes in the MAO-A inhibitory activity (Figure S7E). In this case, close to the *ortho*, *meta* and *para* positions of this ring, a yellow polyhedron is projected, indicating that a hydrophobic substituent may favor the MAO-A inhibitory activity (pIC_{50} > 5.0). Groups such as the methoxy, thiophenyl or 4-bromo-2-thiophenyl follow this tendency, as for compounds **140** to **147**. However, at the outer border of the yellow polyhedron, a gray polyhedron is extended, indicating that a larger hydrophobic substituent may decrease the MAO-A inhibitory activity. This is consistent with the methoxy and methyl substituents of compounds **11**, **41** and **50** (pIC_{50} < 5.0).

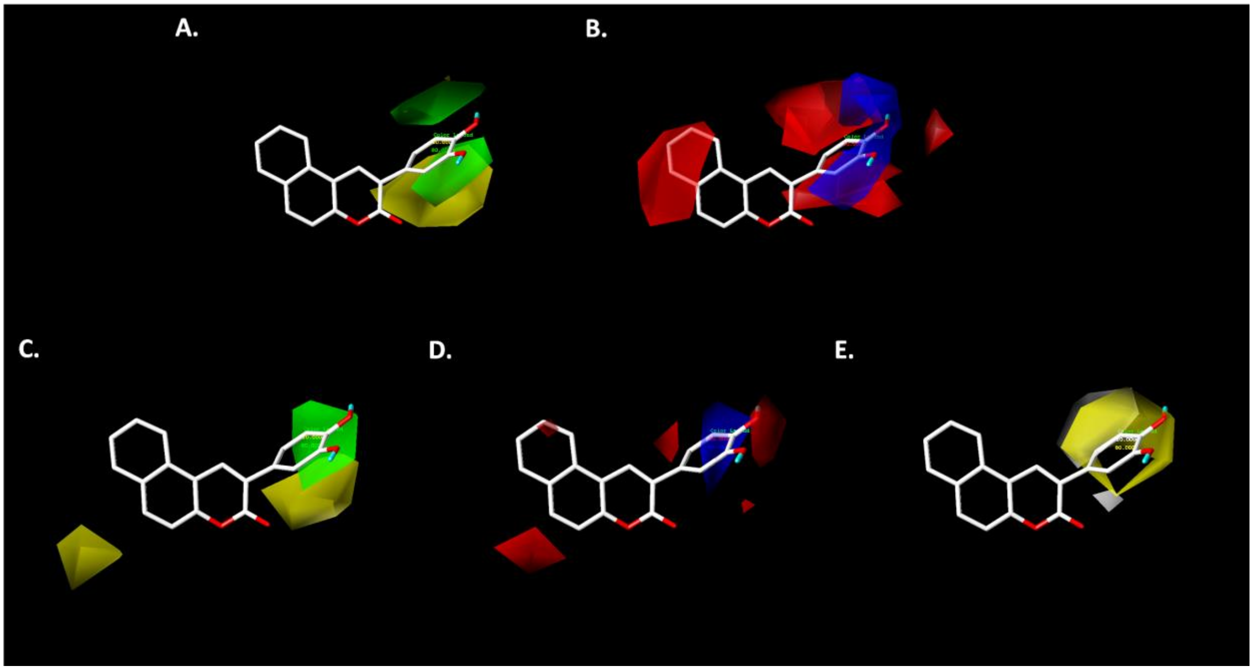

Figure S7. Contour map of 3D-QSAR results for MAO-A inhibitors around the 3-(3',4'-dihydroxyphenyl)benzo[*f*]coumarin (**84**). **A.** CoMFA steric map. **B.** CoMFA electrostatic map. **C.** CoMSIA steric map. **D.** CoMSIA electrostatic map. **E.** CoMSIA hydrophobic map. Color code: steric contour map green – bulky groups are favorable for the activity; steric contour map yellow – small groups are favorable for the activity; electrostatic contour map red – negative charge is favorable for the activity; electrostatic contour map blue – positive charge is favorable for the activity; hydrophobic contour map yellow – hydrophobic groups are favorable for the activity; hydrophobic contour map grey – hydrophilic groups are favorable for the activity.

Docking study on MAO-A with compound 3-(3',4'-dihydroxyphenyl)benzo[f]coumarin

In the case of MAO-A, 3-(3',4'-dihydroxyphenyl)benzo[*f*]coumarin (**84**) has been used for the study, and its affinity energy was calculated as -7.7 Kcal/mol (Figure S8). This figure shows that the aryl group containing the catechol fragment is oriented towards the FAD600, remaining between the Tyr444 and Tyr407 residues. These residues allow the polarization of the ligand to be oxidized [45]. Additionally, the stability of compound **84** is caused by hydrogen-bond interactions between hydroxyl groups and the Asn181 and Tyr197 residues, as well as the polar and hydrophobic interactions with the residues highlighted in Figure S8.

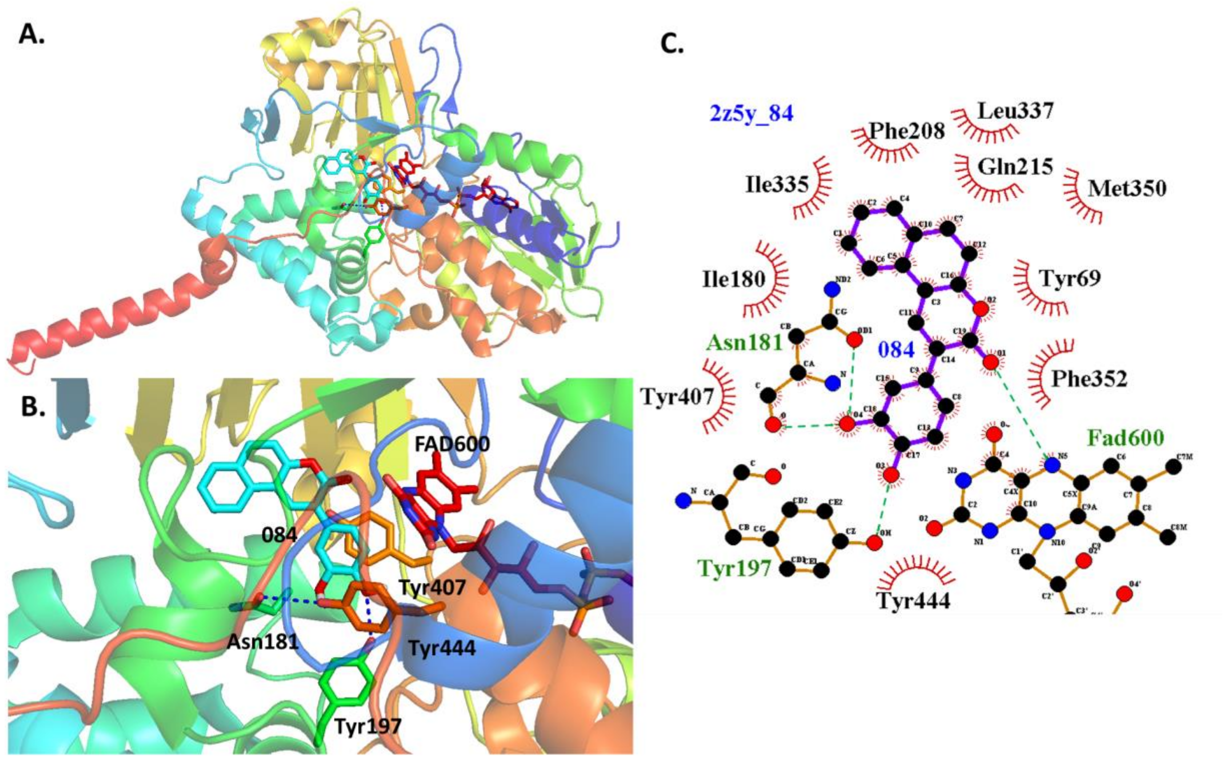

Figure S8. Molecular docking of 3-(3',4'-dihydroxyphenyl)benzo[*f*]coumarin (**84**) on the MAO-A binding pocket. **A.** 3D overview within the MAO-A active site. **B.** Best orientation of compound **84** on the MAO-A active site. **C.** Details of polar, van der Waals and hydrogen-bond interactions established by compound **84** with the residues on the MAO-A active site.

Complementing the information obtained from the molecular coupling analysis (Figure S8) with the contour maps represented in Figure S7, the increase in volume in the catechol area is limited, due to the potential steric impediment that can be generated with the Tyr444 and Tyr407 residues. This situation may cause the decrease or loss of the MAO-A inhibitory activity. On the other hand, the presence of electron-deficient groups close to the hydroxyl groups of the catechol fragment may favor the activity (Figure S7B-D) since these are oriented towards the FAD600, favoring the polar interaction between the ligand **84** and the co-factor. Additionally, the presence of electron-rich substituents close to position 7 of the benzo[*f*]coumarin (red polyhedron, Figure S7B-D), is related to the possible π-cation interaction between the Gln215 residue and the ligand, as well as the perpendicular π-stacking interaction with the Phe208 residue. In the case of the hydrophobic map (Figure S7E), hydrophobic substituents mainly in the meta position of the aryl fragment that

has the catechol function may favor the MAO-A inhibitory activity, consistent with the possible interaction between this fragment and the FAD600 residue.

3D-QSAR results for trans-resveratrol, 3-phenylcoumarin and trans-6-styrylcoumarin

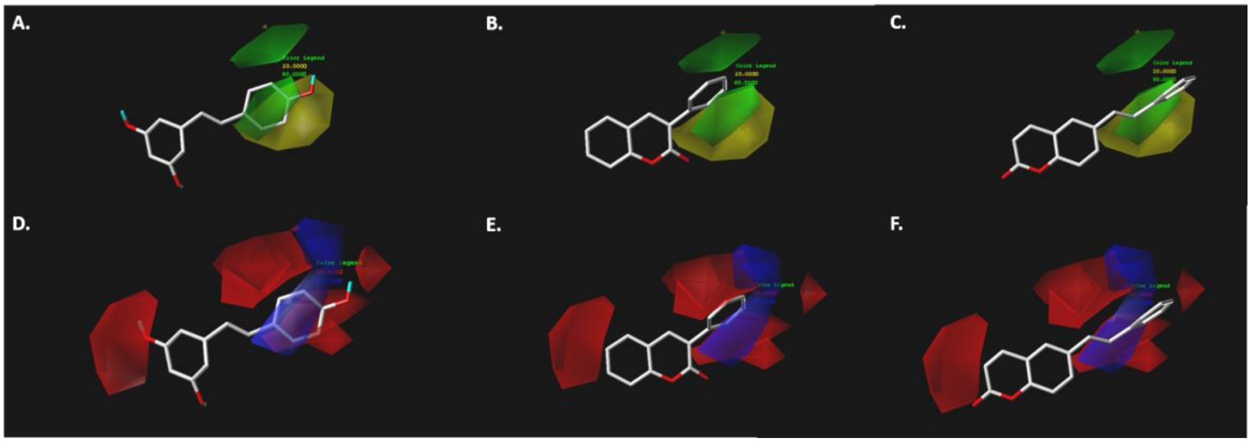

Figure S9. Overlay of *trans*-resveratrol, 3-phenylcoumarin and *trans*-6-styrylcoumarin on the contour maps obtained from the MAO-A CoMFA model. **A.** *trans*-Resveratrol and steric map. **B.** 3-Phenylcoumarin and steric map. **C.** *trans*-6-Styrylcoumarin and steric map. **D.** *trans*-Resveratrol and electrostatic map. **E.** 3-Phenylcoumarin and electrostatic map. **F.** *trans*-6-Styrylcoumarin and electrostatic map. Color code: steric contour map green – bulky groups are favorable for the activity; steric contour map yellow – small groups are favorable for the activity; electrostatic contour map red – negative charge is favorable for the activity; electrostatic contour map blue – positive charge is favorable for the activity; hydrophobic contour map yellow – hydrophobic groups are favorable for the activity; hydrophobic contour map grey – hydrophilic groups are favorable for the activity.

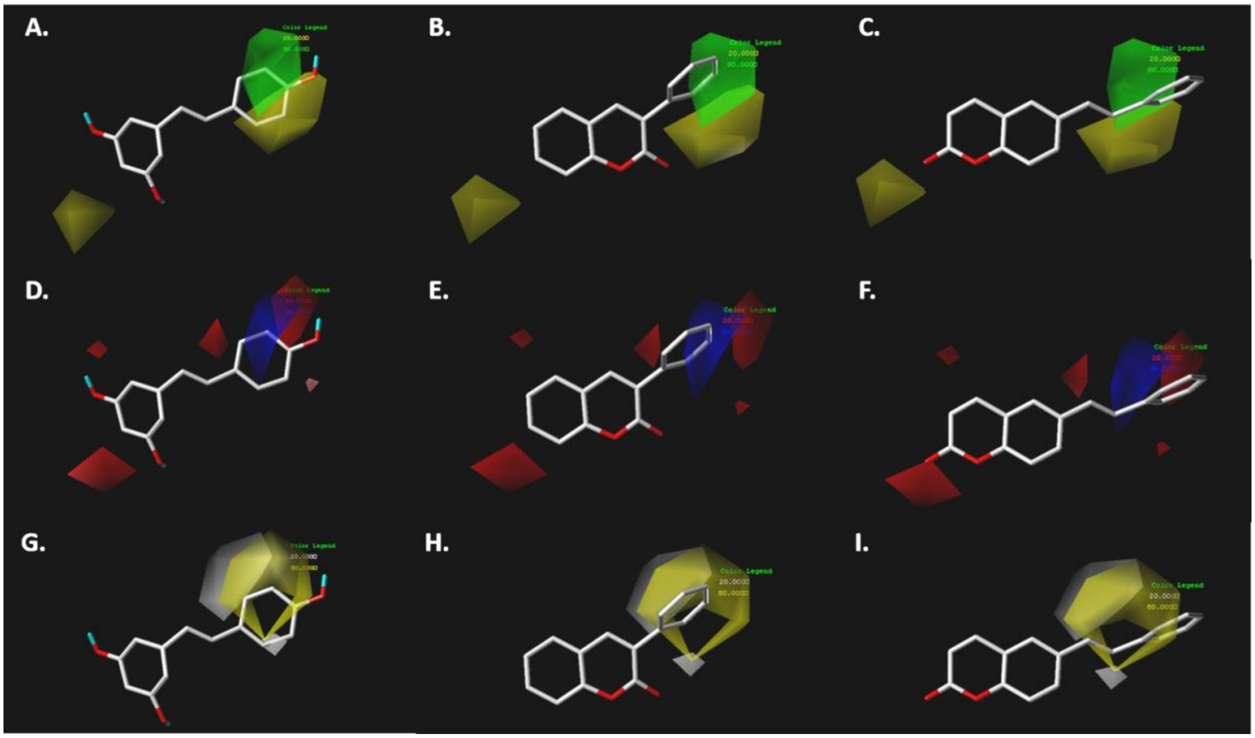

Figure S10. Overlay of *trans*-resveratrol, 3-phenylcoumarin and *trans*-6-styrylcoumarin on the contour maps obtained from the MAO-A CoMSIA model. **A.** *trans*-Resveratrol and steric map. **B.** 3-Phenylcoumarin and steric map. **C.** *trans*-6-Styrylcoumarin and steric map. **D.** *trans*-Resveratrol and electrostatic map. **E.** 3-Phenylcoumarin and electrostatic map. **F.** *trans*-6-Styrylcoumarin and electrostatic map. **G.** *trans*-Resveratrol and hydrophobic map. **H.** 3-Phenylcoumarin and hydrophobic map. **I.** *trans*-6-Styrylcoumarin and hydrophobic map. Color code: steric contour map green – bulky groups are favorable for the activity; steric contour map yellow – small groups are favorable for the activity; electrostatic contour map red – negative charge is favorable for the activity; electrostatic contour map blue – positive charge is favorable for the activity; hydrophobic contour map yellow – hydrophobic groups are favorable for the activity; hydrophobic contour map grey – hydrophilic groups are favorable for the activity.

Molecular dynamics simulations for 3-phenylcoumarin, 3-(3'-bromophenyl)-6-methylcoumarin, trans-resveratrol and trans-6-styrylcoumarin

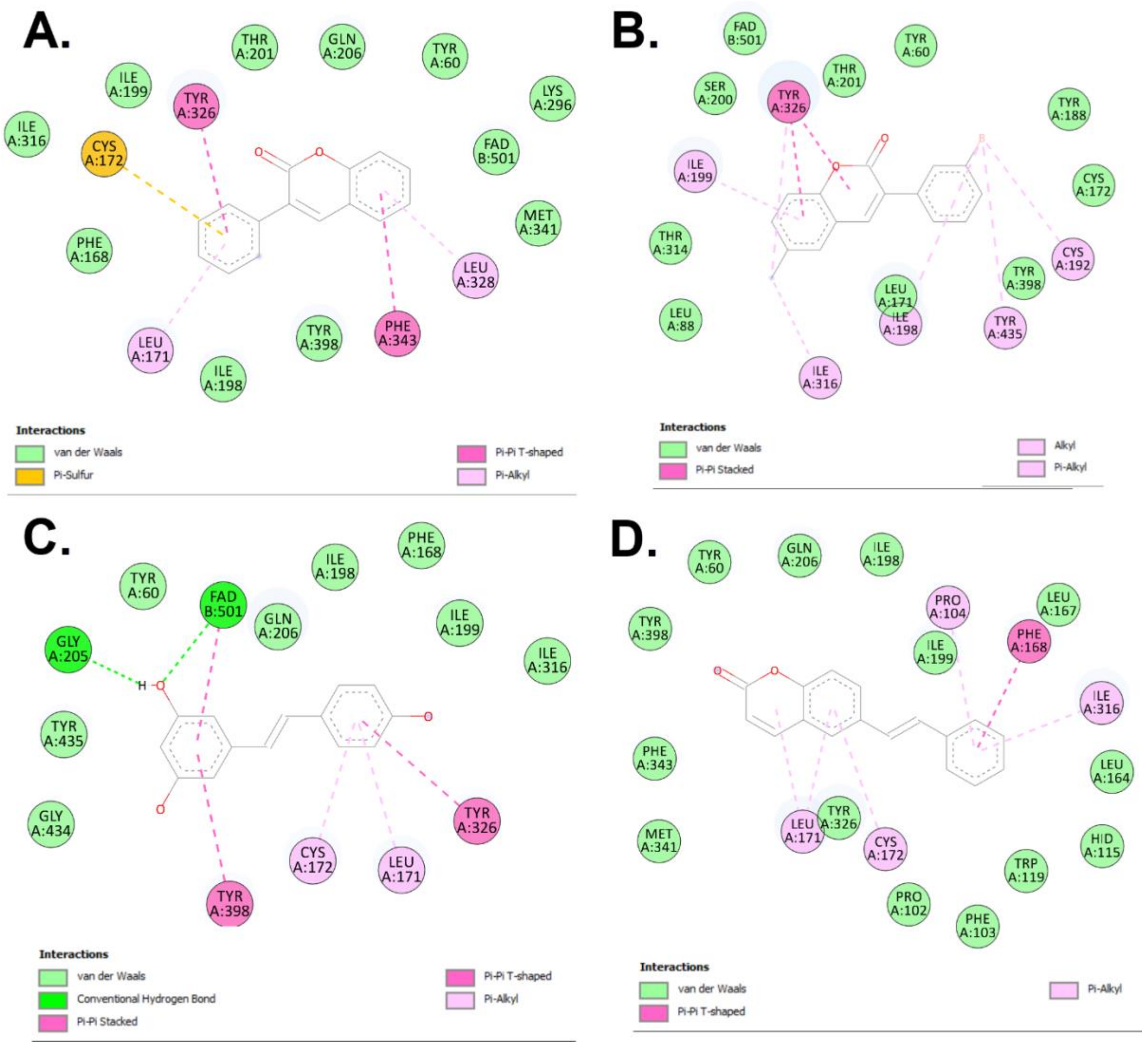

Figure S11. Schematic enzyme-ligand interactions of 3-phenylcoumarin (**A**), 3-(3'-bromophenyl)-6-methylcoumarin (**B**), *trans*-resveratrol (**C**) and *trans*-6-styrylcoumarin (**D**) at active site of MAO-B of 100 ns molecular dynamics simulations.

REFERENCES

1. Matos, M. J.; Viña, D.; Quezada, E.; Picciau, C.; Delogu, G.; Orallo, F.; Santana, L.; Uriarte, E. Regioselective synthesis of different bromo substituted 3-arylcoumarins. *Synthesis* **2010**, *16*, 2763-2766.

2. Matos, M. J.; Santana, L.; Uriarte, E. 3-Phenylcoumarin. *Acta Cryst.* **2012**, *E68*, o2645.

3. Cushman, M.; Nagarathnam, D.; Gopal, D.; Chakraborti, A. K.; Lin, C. M.; Hamel, E. Synthesis and evaluation of stilbene and dihydrostilbene derivatives as potential anticancer agents that inhibit tubulin polymerization. *J. Med. Chem.* **1991**, *34*, 2579-2588.

4. Altomare, A.; Burla, M. C.; Camalli, M.; Cascarano, G.; Giacovazzo, C.; Guagliardi, A.; Moliterni, A. G. G.; Polidori, G.; Spagna, R. SIR97: a new tool for crystal structure determination and refinement. *J. Appl. Cryst.* **1999**, *32*, 115-119.

5. Sheldrick, G. M. SHELXL97. University of Göttingen, Germany, **1997**.

6. Farrugia, L. J. *ORTEP-3* for Windows - a version of ORTEP-III with a Graphical User Interface (GUI). *J. Appl. Cryst.* **1997**, *30*, 565-366.

7. Farrugia, L. J. *WinGX* suite for small-molecule single-crystal crystallography. *J. Appl. Cryst.* **1999**, *32*, 837-838.

8. Mellado, M.; González, C.; Mella, J.; Aguilar, L.F.; Viña, D.; Uriarte, E.; Cuellar, M.; Matos, M.J. Combined 3D-QSAR and docking analysis for the design and synthesis of chalcones as potent and selective monoamine oxidase B inhibitors. *Bioorg. Chem.* **2021**, *108*, 104689.

9. Mellado, M.; Mella, J.; González, C.; Viña, D.; Uriarte, E.; Matos, M.J. 3-Arylcoumarins as highly potent and selective monoamine oxidase B inhibitors: Which chemical features matter? *Bioorg. Chem.* **2020**, *101*, 103964.

10. Apablaza, G.; Montoya, L.; Morales-Verdejo, C.; Mellado, M.; Cuellar, M.; Lagos, C.F.; Soto-Delgado, J.; Chung, H.; Pessoa-Mahana, C.D.; Mella, J. 2D-QSAR and 3D-QSAR/CoMSIA

Studies on a Series of (R)-2-((2-(1H-Indol-2-yl)ethyl)amino)-1-Phenylethan-1-ol with Human β3-Adrenergic Activity. *Molecules*, **2017**, *22*, 404.

11. Xue, C.X.; Cui, S.Y.; Liu, M.C.; Hu, Z.D.; Fan, B.T. 3D QSAR studies on antimalarial alkoxylated and hydroxylated chalcones by CoMFA and CoMSIA. *Eur. J. Med. Chem.* **2004**, *39*, 745-753.

12. Klebe, G.; Abraham, U.; Mietzner, T. Molecular Similarity Indices in a Comparative Analysis (CoMSIA) of Drug Molecules to Correlate and Predict Their Biological Activity. *J. Med. Chem.* **1994**, *37*, 4130-4146.

13. Matos, M.J.; Viña, D.; Quezada, E.; Picciau, C.; Delogu, G.; Orallo, F.; Santana, L.; Uriarte, E. A new series of 3-phenylcoumarins as potent and selective MAO-B inhibitors. *Bioorg. Med. Chem. Lett.* **2009**, *19*, 3268-3270.

14. Matos, M.J.; Viña, D.; Janeiro, P.; Borges, F.; Santana, L.; Uriarte, E. New halogenated 3-phenylcoumarins as potent and selective MAO-B inhibitors. *Bioorg. Med. Chem. Lett.* **2010**, *20*, 5157-5160.

15. Matos, M.J.; Terán, C., Pérez-Castillo, Y.; Uriarte, E.; Santana, L.; Viña, D. Synthesis and Study of a Series of 3-Arylcoumarins as Potent and Selective Monoamine Oxidase B Inhibitors. *J. Med. Chem.* **2011**, *54*, 7127-7137.

16. Serra, S.; Ferino, G.; Matos, M.J.; Vázquez-Rodríguez, S.; Delogu, G.; Viña, D.; Cadoni, E.; Santana, L.; Uriarte, E. Hydroxycoumarins as selective MAO-B inhibitors. *Bioorg. Med. Chem. Lett.* **2012**, *22*, 258-261.

17. Viña, D.; Matos, M.J.; Ferino, G.; Cadoni, E.; Laguna, R.; Borges, F.; Uriarte, E.; Santana, L. 8-Substituted 3-Arylcoumarins as Potent and Selective MAO-B Inhibitors: Synthesis, Pharmacological Evaluation, and Docking Studies. *ChemMedChem*, **2012**, *7*, 464-470.

18. Viña, D.; Matos, M.J.; Yáñez, M.; Santana, L.; Uriarte, E. 3-Substituted coumarins as dual inhibitors of AChE and MAO for the treatment of Alzheimer's disease. *MedChemComm*, **2012**, *3*, 213-218.

19. Ferino, G.; Cadoni, E.; Matos, M.J.; Quezada, E.; Uriarte, E.; Santana, L.; Vilar, S.; Tatonetti, N.P.; Yáñez, M.; Viña, D.; Picciau, C.; Serra, S.; Delogu, G. MAO inhibitory activity of 2-arylbenzofurans versus 3-arylcoumarins: synthesis, in vitro study, and docking calculations. *ChemMedChem*, **2013**, *8*, 956-966.

20. Matos, M.J.; Vilar, S.; Gonzalez-Franco, R.M.; Uriarte, E.; Santana, L.; Friedman, C.; Tatonetti, N.P.; Viña, D.; Fontenla, J.A. Novel (coumarin-3-yl)carbamates as selective MAO-B inhibitors: synthesis, in vitro and in vivo assays, theoretical evaluation of ADME properties and docking study. *Eur. J. Med. Chem*. **2013**, *63*, 151-161

21. Matos, M.J.; Vilar, S.; García-Morales, V.; Tatonetti, N.P.; Uriarte, E.; Santana, L.; Viña, D. Insight into the Functional and Structural Properties of 3-Arylcoumarin as an Interesting Scaffold in Monoamine Oxidase B Inhibition. *CemMedChem*, **2014**, *9*, 1488-1500.

22. Matos, M.J.; Janeiro, P.; González-Franco, R.M.; Vilar, S.; Tatonetti, N.P.; Santana, L.; Uriarte, E.; Borges, F.; Fontenla, J.A.; Viña, D. Synthesis, pharmacological study and docking calculations of new benzo[f]coumarin derivatives as dual inhibitors of enzymatic systems involved in neurodegenerative diseases. *Future Med. Chem*. **2014**, *6*, 371-383.

23. Matos, M.J.; Rodríguez-Enríquez, F.; Vilar, S.; Santana, L.; Uriarte, E.; Hripcsak, G.; Estrada, M.; Rodríguez-Franco, M.I., Viña, D. Potent and selective MAO-B inhibitory activity: amino- versus nitro-3-arylcoumarin derivatives. *Bioorg. Med. Chem. Lett.* **2015**, *25*, 642-648.

24. Matos, M.J.; Rodríguez-Enríquez, F.; Borges, F.; Santana, L.; Uriarte, E.; Estrada, M.; Rodríguez-Franco, M.I.; Laguna, R.; Viña, D. 3-Amidocoumarins as Potential Multifunctional Agents against Neurodegenerative Diseases. *ChemMedChem*, **2015**, *10*, 2071-2079.

25. Fonseca, A.; Matos, M.J.; Reis, J.; Duarte, Y.; Gutiérrez, M.; Santana, L.; Uriarte, E.; Borges, F. Exploring coumarin potentialities: development of new enzymatic inhibitors based on the 6-methyl-3-carboxamidocoumarin scaffold. *RSC Advances*, **2016**, *6*, 49764-49768.

26. Fonseca, A.; Reis, J.; Silva, T.; Matos, M.J.; Bagetta, D.; Ortuso, F.; Alcaro, S.; Uriarte, E.; Borges, F. Coumarin versus Chromone Monoamine Oxidase B Inhibitors: Quo Vadis? *J. Med. Chem.* **2017**, *60*, 7206-7212.

27. Rodríguez-Enríquez, F.; Viña, D.; Uriarte, E.; Fontenla, J.A.; Matos, M.J. Discovery and optimization of 3-thiophenylcoumarins as novel agents against Parkinson's disease: Synthesis, *in vitro* and *in vivo* studies. *Bioorg. Chem.* **2020**, *101*, 103986.

28. Matos, M.J., Herrera Ibatá, D.M.; Uriarte, E.; Viña, D. Coumarin-Rasagiline Hybrids as Potent and Selective hMAO-B Inhibitors, Antioxidants, and Neuroprotective Agents. *ChemMedChem*, **2020**, *15*, 532-538.

29. Rodríguez-Enríquez, F.; Viña, D.; Uriarte, E.; Laguna, R.; Matos, M.J. 7-Amidocoumarins as Multitarget Agents against Neurodegenerative Diseases: Substitution Pattern Modulation. *ChemMedChem*, **2021**, *16*, 179-186.

30. Clark, M.; Cramer, R.D.; Van Opdenbosch, N. Validation of the general purpose tripos 5.2 force field. *J. Comp. Chem*. **1989**, *10*, 982-1012.

31. Golbraikh, A.; Tropsha, A. Beware of q^2! *J. Mol. Graph. Modell*. **2002**, *20*, 269-276.

32. Tropsha, A. Best Practices for QSAR Model Development, Validation, and Exploitation. *Mol. Inform*. **2010**, *29*, 476-488.

33. Roy, K.; Chakraborty, P.; Mitra, I.; Kumar Ojha, P.; Kar, S.; Narayan Das, R. Some case studies on application of "rm2" metrics for judging quality of quantitative structure–activity relationship predictions: Emphasis on scaling of response data. *J. Comp. Chem.* **2013**, *34*, 1071-1082.

34. Roy, K.; Kar, S.; Ambure, P. On a simple approach for determining applicability domain of QSAR models. *Chemometr. Intell. Lab.* **2015**, *145*, 22-29.

35. Son, S-Y.; Ma, J.; Kondou, Y.; Yoshimura, M.; Yamashita, E.; Tsukihara, T. Structure of human monoamine oxidase A at 2.2-Å resolution: The control of opening the entry for substrates/inhibitors. *P. Natl. Acad. Sci. USA*. **2008**, *105*, 5739-5744.

36. Binda, C.; Newton-Vinson, P.; Hubálek, F.; Edmonson, D.E.; Mattevi, A. Structure of human monoamine oxidase B, a drug target for the treatment of neurological disorders. *Nat. Struct. Biol.*

2002, *9*, 22-26.

37. Trott, O.; Olson, A.J. AutoDock Vina: Improving the speed and accuracy of docking with a new scoring function, efficient optimization, and multithreading. *J. Comp. Chem.* **2010**, *31*, 455-461.

38. L. Schrödinger, The PyMOL Molecular Graphics System, Schrödinger, LLC, 2003-2017.

39. Laskowski, R.A.; Swindells, M.B. LigPlot+: Multiple Ligand–Protein Interaction Diagrams for Drug Discovery. *J. Chem. Inf. Model.* **2011**, 51, *10*, 2778-2786.

40. Wallace, A.C.; Laskowski, R.A.; Thornton, J.M. LIGPLOT: a program to generate schematic diagrams of protein-ligand interactions. *Protein Eng. Des. Sel.* **1995**, *8*, 127-134.

41. Abraham, M.J.; Murtola, T.; Schulz, R.; Páll, S.; Smith, J.C.; Hess, B.; Lindahl, E. GROMACS: High performance molecular simulations through multi-level parallelism from laptops to supercomputers. SoftwareX **2015**, *1*, 19-25.

42. Jo, S.; Kim, T.; Iyer, V.G.; Im, W. CHARMM-GUI: a web-based graphical user interface for CHARMM. *J. Comput. Chem.* **2008**, *29(11)*, 1859-1865.

43. Huang, J.; Rauscher, S.; Nawrocki, G.; Ran, T.; Feig, M.; De Groot, B.L.; Grubmüller, H.; MacKerell, A.D. CHARMM36m: an improved force field for folded and intrinsically disordered proteins. *Nat. methods* **2017**, *14(1)*, 71-73.

44. Kumari, R.; Kumar, R. Open Source Drug Discovery Consortium, & Lynn, A. g_mmpbsa A GROMACS tool for high-throughput MM-PBSA calculations. *J. Chem. Inf. Model.* **2014**, *54(7)*, 1951-1962.

45. Abad, E.; Zenn, R.K.; Kästner, J. Reaction Mechanism of Monoamine Oxidase from QM/MM Calculations. *J. Phys. Chem. B* **2013**, *117(46)*, 14238-14246.
